# Unifying approaches from statistical genetics and phylogenetics for mapping phenotypes in structured populations

**DOI:** 10.1371/journal.pbio.3002847

**Published:** 2024-10-09

**Authors:** Joshua G. Schraiber, Michael D. Edge, Matt Pennell

**Affiliations:** 1 Department of Quantitative and Computational Biology, University of Southern California, Los Angeles, California, United States of America; 2 Department of Biological Sciences, University of Southern California, Los Angeles, California, United States of America; Indiana University, UNITED STATES OF AMERICA

## Abstract

In both statistical genetics and phylogenetics, a major goal is to identify correlations between genetic loci or other aspects of the phenotype or environment and a focal trait. In these 2 fields, there are sophisticated but disparate statistical traditions aimed at these tasks. The disconnect between their respective approaches is becoming untenable as questions in medicine, conservation biology, and evolutionary biology increasingly rely on integrating data from within and among species, and once-clear conceptual divisions are becoming increasingly blurred. To help bridge this divide, we lay out a general model describing the covariance between the genetic contributions to the quantitative phenotypes of different individuals. Taking this approach shows that standard models in both statistical genetics (e.g., genome-wide association studies; GWAS) and phylogenetic comparative biology (e.g., phylogenetic regression) can be interpreted as special cases of this more general quantitative-genetic model. The fact that these models share the same core architecture means that we can build a unified understanding of the strengths and limitations of different methods for controlling for genetic structure when testing for associations. We develop intuition for why and when spurious correlations may occur analytically and conduct population-genetic and phylogenetic simulations of quantitative traits. The structural similarity of problems in statistical genetics and phylogenetics enables us to take methodological advances from one field and apply them in the other. We demonstrate by showing how a standard GWAS technique—including both the genetic relatedness matrix (GRM) as well as its leading eigenvectors, corresponding to the principal components of the genotype matrix, in a regression model—can mitigate spurious correlations in phylogenetic analyses. As a case study, we re-examine an analysis testing for coevolution of expression levels between genes across a fungal phylogeny and show that including eigenvectors of the covariance matrix as covariates decreases the false positive rate while simultaneously increasing the true positive rate. More generally, this work provides a foundation for more integrative approaches for understanding the genetic architecture of phenotypes and how evolutionary processes shape it.

## Abstract 2

Statistical genetics and phylogenetics have different methods of finding associations with phenotypes while controlling for ancestry. This paper shows that the standard approaches in these fields are actually special cases of the same general approach, enabling more integrative future studies.

## 1 Introduction

Statistical genetics and phylogenetic comparative biology share the goal of identifying correlations between features of individuals (or populations) that share ancestry. In the case of statistical genetics, researchers search for causal genetic variants underlying a phenotype of interest, whereas in phylogenetic comparative biology, researchers are typically interested in testing for associations among phenotypes or between a phenotype and an environmental variable. In both cases, these tests are designed to isolate the influence of a focal variable from that of many potential confounding variables. But despite the shared high-level goal, the statistical traditions in these 2 fields have developed largely separately, and—at least superficially—do not resemble each other. Moreover, researchers in these 2 statistical traditions may have different understandings of the nature of the problems they are trying to solve.

In statistical genetics, phenotypes and genotypes can be spuriously associated because of confounding due to population structure [1–4] or assortative mating [5,6]. For example, in their famous “chopsticks” thought experiment, Lander and Schork [1] pointed out that genetic variants that have drifted to higher frequency in subpopulations in which chopsticks are frequently used will appear, in a broad sample, to be associated with individual ability to use chopsticks, even though the association is due to cultural confounding and not to genetic causation. Confounding can also be genetic [7]—if a genetic variant that changes a phenotype is more common in one population than others, leading to differences in average phenotype among populations, then other, noncausal variants that have drifted to relatively high frequency in this population may appear to be associated with the phenotype in a broad sample. In addition to affecting genome-wide association study (GWAS) results, such confounding can affect heritability estimation [8,9], genetic correlation estimates [10,11], and prediction of phenotypes from polygenic scores [12–16]. Although many candidate solutions have been offered [17–21], the 2 most common approaches involve adjusting for shared ancestry using the genetic relatedness matrix (GRM, [22]), either by incorporating individual values on the first several eigenvectors of this matrix (i.e., the principal components of the genotype matrix) as fixed effects [23], or by modeling covariance among individuals attributable to genome-wide relatedness in a linear mixed model (LMM, [24–28]).

In phylogenetic comparative biology, researchers typically aim to control for the similarity of related species by incorporating the species tree into the analysis. There has been a great deal of controversy as to what the underlying goals and implicit assumptions of phylogenetic comparative methods (PCMs) are (see for examples refs. [29–36]). But broadly speaking, it seems that many researchers understand the goal of PCMs to be avoiding “phylogenetic pseudoreplication” [37]—mistaking similarity due to shared phylogenetic history for similarity due to independent evolutionary events [34]. This is most commonly done by conducting a standard regression, using either generalized least squares (GLSs) or a generalized linear mixed model (GLMM), but including the expected covariance structure owing to the phylogeny [38–42]. (Throughout this paper, we do not make a distinction between phylogenetic GLS and phylogenetic GLMM models. We refer to them generically by the shorthand GLS for the general case and PGLS for cases where the phylogenetic variance-covariance matrix is used.) This covariance structure reflects both the relatedness of species and the expected distribution of phenotypes under a model of phenotypic evolution [43,44], such as a Brownian motion [45] and related alternatives [44]. (The “phylogenetically independent contrasts” method [46], which ushered in modern PCMs, is statistically equivalent to a PGLS model assuming a Brownian model [47].)

In recent years, however, signs have emerged that these 2 subfields may benefit from closer conversation, as emerging approaches in both statistical genetics and phylogenetics encounter questions that call for the other subfield’s expertise. For example, in humans, evolutionarily conserved sequences are enriched for trait and disease heritability [48,49], and conservation across related species can be used to prioritize medically relevant variants in fine mapping [50,51] and rare-variant association studies [52,53]. Similarly, multispecies alignments are now used by conservation geneticists to estimate the fitness effects of mutations in wild populations [54,55] and by plant breeders to aid in genomic selection [56,57]. And there is growing interest in using estimated ancestral recombination graphs (ARGs) to perform explicitly tree-based versions of QTL mapping and complex trait analysis [58,59]. From the phylogenetics side, researchers are increasingly employing GWAS-like approaches (“PhyloG2P” methods; [60]) for mapping phenotypes of interest for which the variation primarily segregates among rather than within species.

Such emerging connections suggest that it would be beneficial to understand the ways in which statistical genetics and phylogenetic comparative biology relate to each other. Here, we show that methods in these 2 fields can be understood as closely related special cases of the same more general model. In Section 2.1, we start from first principles and develop a general statistical model for investigating associations between focal variables while controlling for shared ancestry. Then, in Section 2.2, we outline how this general model specializes to the settings of GWAS by assuming genotypes and effect sizes are conditionally independent (Section 2.2.1); animal breeding by assuming known pedigree relationships (Section 2.2.2); expected relatedness given a fixed coalescent tree (Section 2.2.3); and phylogenetics given a fixed species tree (Section 2.2.4). Next, in Section 2.3, we provide both theoretical (Section 2.3.1) and simulation-based (Section 2.3.2) demonstrations of when and how different commonly used approaches to controlling the effects of population structure succeed and fail on different timescales. Finally, in Section 2.4 we show an application of a commonly used tool of statistical genetics in a phylogenetic setting to demonstrate the utility of understanding the connections between these methodological traditions.

## 2 Results

### 2.1. A standard model for a quantitative trait

We assume a standard model in which many genetic factors of small effect influence a phenotype in an additive way—that is, there is no dominance or interaction among genetic loci (epistasis). Denoting by *β*_*l*_ the additive effect size of the variant at the *l*th locus and *G*_*il*_ the genotype of the *i*th individual at the *l*th locus, under this model,

$$A_{i}=\sum_{l}\;\beta_{l}G_{il},$$ (1)

where *A*_*i*_ is the genetic component of the phenotype of individual *i*, sometimes called a genetic value or breeding value. We then express the phenotype of individual *i*, denoted *Y*_*i*_, as the sum of the genetic component and an environmental component, *E*_*i*_:

$$Y_{i}=A_{i}+E_{i}.$$ (2)

Due to shared ancestry, the genotypes of individuals in the sample will be correlated and thus, the genetic components of the individuals in the sample will be correlated. Moreover, the environments experienced by individuals may be correlated, and these environmental effects may be correlated with the genetic components. If we are interested in understanding the factors that shape the trait of interest, we must control for the covariance induced by shared genetics and shared environment. This covariance can be written as follows:

$$Cov(Y_{i},Y_{j})=Cov(A_{i},A_{j})+Cov(A_{i},E_{j})+Cov(E_{i},A_{j})+Cov(E_{i},E_{j}).$$ (3)

For the rest of the paper, our focus will be on the first term, Cov(*A*_*i*_, *A*_*j*_), the covariance in phenotypes between individuals due to genetic covariance. We focus on this term because, as we show subsequently, many models used by both statistical geneticists and phylogenetic biologists can be understood without reference to the components that include environmental effects. There are some circumstances in which genetic covariance in Eq 6 is undefined, such as when effect sizes have an undefined variance [61], or under certain phenomenological models of evolution on phylogenies [62,63]; we reserve these situations for future work and focus on situations in which the genetic covariance is finite in the subsequent sections.

### 2.2. Conceptualizations of the genetic covariance among individuals

Individuals who are more closely related will have more similar genotypes. For example, individuals in the same local population may share the same alleles identical by descent due to recent common ancestry. On the other hand, individuals in different species may not share alleles due to the species being fixed for alternative alleles at a given locus. Using Eq 1,

$$Cov(A_{i},A_{j})=Cov(\sum_{l}\beta_{l}G_{il},\sum_{l}\beta_{l}G_{jl})$$

$$=\sum_{l}\;Cov(\beta_{l}G_{il},\beta_{l}G_{jl})+\sum_{l\neq k}\;Cov(\beta_{l}G_{il},\beta_{k}G_{jk}).$$ (4)

The first term arises from the correlations between individuals at single loci, whereas the second term arises from correlations among loci between individuals. We focus on the first term, and all derivations below assume the second term is equal to zero, despite the fact that it will generally not be identically zero in realistic situations. As with gene-environment correlation in the previous section, many conceptualizations of genetic covariance used in practice can be viewed as neglecting the second term. Under a neutral model, the second term is 0 in expectation over distinct realizations of the evolutionary process, and its variance does not grow with the number of loci under commonly studied forms of population structure [64,65]. Intuitively, this term disappears in expectation under neutral evolution because the effect sizes and genotypes are uncorrelated, and hence the sum is of a mix of positive and negative terms, which cancel out on average, although it will likely be non-zero in any particular data set. Nonetheless, the second term in Eq 4 will often be nonzero in practice, and systematic correlations among loci that make the term nonzero in expectation can arise in biologically realistic situations, for example, if directional selection acts on polygenic traits. If a population experiences directional selection on a highly polygenic phenotype, much of the phenotypic change, compared with a related population that has not experienced such selection, is due to to small, coordinated changes in allele frequency, leading to systematic covariances among loci, even if they are unlinked [65,66]. Although we do not discuss these complications here, linkage can affect the evolution of polygenic traits [67] and the results of heritability estimates [68].

We would like to understand how different assumptions about the evolutionary process affect the genetic covariance among individuals. To do so, it is necessary to make further assumptions about the effect sizes and genotypes. In principle, different modeling scenarios might require us to cast as random the genotypes (e.g., because they are the outcome of mating and mendelian processes that are viewed as random), the effect sizes (e.g., because they arise due to random mutations on a haplotype or are unknown), or both. (Either genotypes or effect sizes might reasonably be—and sometimes are—treated as fixed and not random in some scenarios, as we discuss below. Our formulation of random effect sizes is distinct from that of Kempthorne [69], who defined allelic effects that can depend on allele frequency and other factors that affect the trait [70].) Moreover, the relationship between effect size and genotype can depend on details of the underlying evolutionary model. Hence, in general, $Cov(\beta_{l}G_{il},\beta_{l}G_{jl})\neq \beta_{l}^{2}Cov(G_{il},G_{jl})$.

Nonetheless, by making assumptions about the evolutionary process, we can obtain useful approximations of the genetic covariance. As an example, developed further below, consider a model in which mutation and selection act on a quantitative trait. The effect size of a locus, *β*_*l*_, might be modeled as being drawn from a distribution, and its allele frequency *p*_*l*_ then could evolve according to a model that depends on *β*_*l*_. Then, genotypes *G*_*il*_ and *G*_*jl*_ are drawn according to allele frequency and possibly other features. In this scenario generally, the relationship between *β*_*l*_ and *G*_*il*_ may be complicated. However, if selection is sufficiently weak as not to disrupt Hardy–Weinberg proportions or linkage equilibrium, then genotype frequencies depend only on the allele frequency, *p*_*l*_. In that case, we might represent the situation with a simplified causal graph *β*_*l*_→*p*_*l*_→*G*_*il*_, in which *β*_*l*_ and *G*_*il*_ are conditionally independent given the allele frequency *p*_*l*_ [71–73].

We generalize this notion by considering that in certain cases, like the one discussed above, there may be a latent variable that renders the genotypes *G*_*il*_ and effect sizes *β*_*l*_ conditionally independent. We use *Z* to represent such a variable in general. Conditioning on *Z* and using the definition of covariance and the law of total expectation, the first term becomes

$$Cov(\beta_{l}G_{il},\beta_{l}G_{jl})=E(\beta_{l}^{2}G_{il}G_{jl})-E(\beta_{l}G_{il})E(\beta_{l}G_{jl})$$

$$=E_{Z}(E(\beta_{l}^{2}|Z)E(G_{il}G_{jl}|Z))-E_{Z}(E(\beta_{l}|Z)E(G_{il}|Z))E_{Z}(E(\beta_{l}|Z)E(G_{jl}|Z))$$ (5)

This formula applies as long as the genetic covariance exists and the evolutionary model admits a variable *Z* that accounts for the relationship between effect sizes and genotypes (and all the relevant expectations exist). Moreover, it applies when the variable *Z* = *β* or *Z* = *G*.

Below, we will explore how applications across statistical and evolutionary genetics specialize Eq 5 in different ways to create a matrix summarizing genetic covariance relevant to phenotypic variation, which we refer to as Σ. In a sample of *n* individuals (or *n* species), Σ is *n* × *n*, and Σ_*ij*_ is proportional to some version of Eq 5. We will see that assumptions made in different fields relate to the underlying evolutionary process shaping genetic and phenotypic variation. Among other names, in different settings, Σ might take the form of a “genetic relatedness matrix,” “kinship matrix,” “expected genetic relatedness matrix,” or “phylogenetic variance-covariance matrix.” Below, we consider the off-diagonal entries of each of these matrices in turn.

#### 2.2.1. The genetic relatedness matrix

In this subsection, we show how the general polygenic model described above can yields the GRM, a realization of Σ that is commonly used to estimate heritability from SNP data [74] or to accommodate covariance due to relatedness in GWAS [24–26,28]. In statistical-genetic practice, genotypes are typically mean centered, meaning that genotypes are represented as ${\tilde{G}}_{il}=G_{il}-\frac{1}{n}\sum_{j}\;G_{jl}$, and also standardized by $\sqrt{2p_{l}(1-p_{l})}$. With this notation, the *ij*th entry of the canonical GRM is given by

$$\sum_{l}\frac{{\tilde{G}}_{il}}{\sqrt{2p_{l}(1-p_{l})}}\frac{{\tilde{G}}_{jl}}{\sqrt{2p_{l}(1-p_{l})}}=\sum_{l}\frac{{\tilde{G}}_{il}{\tilde{G}}_{jl}}{2p_{l}(1-p_{l})}.$$ (6)

Historically, it seems that the motivation for this form of the GRM was not to characterize covariance in the genetic components of traits. Instead, early uses of the GRM tend to justify the normalization in terms of giving each locus “equal weight,” since under Hardy–Weinberg equilibrium, the variance of ${\tilde{G}}_{il}$ is 2*p*_*l*_(1−*p*_*l*_), or in terms of the fact that the variance in allele frequency change due to one generation of drift is proportional to 2*p*_*l*_(1−*p*_*l*_) [23,75]. However, we show that Eq 6 can also be justified as being proportional to the covariance of the genetic component of the phenotypes of individuals *i* and *j* under certain assumptions.

We begin by setting *Z* = *p*_*l*_ in Eq 5. This move is justified because, under a simplified model of polygenic selection and assuming genotypes are in Hardy–Weinberg proportions, the effect sizes and genotypes are conditionally independent given the allele frequencies [71], and it yields

$$Cov(\beta_{l}{\tilde{G}}_{il},\beta_{l}{\tilde{G}}_{jl})=E_{p}(E(\beta_{l}^{2}|p_{l})E({\tilde{G}}_{il}{\tilde{G}}_{jl}|p_{l})).$$

(The second term in Eq 5 vanishes because mean-centering of genotypes guarantees that $E({\tilde{G}}_{il})=E({\tilde{G}}_{jl})=0.$)

With observed genotypes, the expectation over genotypes at a given frequency can be approximated as follows:

$$E({\tilde{G}}_{il}{\tilde{G}}_{jl}|p_{l}=p)\approx \frac{1}{n_{p}}\sum_{l:p_{l}=p}{\tilde{G}}_{il}{\tilde{G}}_{jl},$$

where *n*_*p*_ is the number of sites with frequency *p*. To obtain the GRM as in Eq 6, we assume that $E(\beta_{l}^{2}|p_{l}=p)\equiv \beta^{2}(p)$ has the form

$$\beta^{2}(p)=\frac{\sigma^{2}}{2p(1-p)},$$ (7)

where *σ*^2^ is simply a constant that we will show is related to the additive genetic variance. Then,

$$Cov(A_{i},A_{j})\approx \sum_{p}\;n_{p}\frac{\sigma^{2}}{2p(1-p)}\frac{1}{n_{p}}\sum_{l:p_{l}=p}\;{\tilde{G}}_{il}{\tilde{G}}_{jl}$$ (8)

$$=\sigma^{2}\sum_{l}\frac{{\tilde{G}}_{il}{\tilde{G}}_{jl}}{2p(1-p)}.$$ (9)

where the final line is equivalent to Eq 6. In Section A in S1 Text, we include a demonstration that Eq 7 arises under a model of mutation-selection balance under Gaussian stabilizing selection on the focal trait, such that

$$\beta^{2}(p)=\frac{2\mu V_{s}}{p(1-p)},$$

where *μ* is the mutation rate at that locus and *V*_*s*_ is the variance of the Gaussian fitness function. This derivation suggests that Eq 7 may be suitable for variants of large effect impacting traits under strong selection, but may not be appropriate when effect sizes are smaller, traits evolve under weaker selection, when there is a substantial contribution of genetic drift [71,76] or when the model is violated in other ways, including when there are multiple traits under stabilizing selection, and the causal variants are pleiotropic for these traits [71], or when alleles’ frequencies are due not to their causal effects on the trait but instead to their LD with causal variants.

This formulation of the GRM also allows estimation of the additive genetic variance, *V*_*A*_, via estimation of *σ*^2^. For a panmictic population with an effect size-allele frequency relationship specified by (7), it can be shown that

$$V_{A}=\sigma^{2}L,$$

where *L* is the number of loci. However, using this approach to estimate the additive genetic variance and heritability may result in errors if the true relationship between allele frequency and effect size is weaker than supposed here. One approach to generalizing the standard GRM is to instead suppose $\beta^{2}(p)\propto (2p(1-p){)}^{-\alpha }$, known as the “*α* model” or “LDAK model” [22,68]. The *α* parameter is often interpreted as related to the strength of selection acting on a trait [76,77]. In plant and animal breeding, sometimes the same normalization is used as in human genetics, and sometimes genotypes are mean centered but not standardized [78–80].

#### 2.2.2. The (pedigree-based) kinship matrix

Historically, plant and animal breeders, along with human and behavior geneticists interested in resemblance of relatives, have frequently faced a situation in which they had: (1) (at least partial) pedigree data describing the parentage of sets of individual plants or animals; (2) phenotypic data on those individuals; but (3) no genome-wide genetic information. In such a situation, one can model the entries of Σ as a function of expected genetic similarity based on the pedigree information, as opposed to realized genetic sharing observed from genotypes [79,81–83]. One can specialize Eq 4 by fixing the effect sizes, leading to

$$Cov(\sum \beta_{l}G_{il},\sum \beta_{l}G_{jl})=2\theta_{ij}V_{A},$$ (10)

where *θ*_*ij*_ is the kinship coefficient (obtained from the pedigree) relating individuals *i* and *j*, and *V*_*A*_ is the additive genetic variance. Although many derivations exist in standard texts (e.g., [83,84]), we include one in Section B in S1 Text for completeness.

Methods based on this formulation include the “animal model” [81,83,85,86], a widely used approach for prediction of breeding values in quantitative genetics. The connection between the animal model and genome-wide marker-based approaches was plain to the quantitative geneticists who first developed marker-based approaches to prediction [78], and it is also noted in papers aimed at human geneticists [22,74,87], whose initial interest in the framework focused on heritability estimation. Similarly, the animal model is known to be intimately connected to the phylogenetic methods we discuss later [40–42]. One implication is that close connections between methods used in statistical genetics and phylogenetics, which are our focus here, must exist.

#### 2.2.3. The expected genetic relatedness matrix (eGRM)

If neither genotypes nor pedigrees are available, additional assumptions are necessary to compute the genetic contribution to phenotypic covariance between individuals. In particular, we let *Z* = *β*, the effect size itself, in Eq 5. Unlike in the previous subsection, the effect sizes are not fixed; they are random and independent of genotype. Effect sizes and genotypes are independent when the focal trait is selectively neutral (and loci are not pleiotropic for or in LD with causal variants for other traits under selection). In this case, one can use a coalescent approach to integrate over alternative realizations of the gene tree(s) and of the mutational process (as in the branch-based approach in [88]). We show in Section C in S1 Text that, when averaged over the mutational histories and gene trees at *L* independent segregating variants,

$$Cov(A_{i},A_{j})=LE(\beta^{2})\frac{E(T_{ij})}{E(T)}.$$ (11)

In principle, the entries of the relatedness matrix could be computed on the basis of a demographic model; in this approach, one would average over both random gene trees and random mutations. This is the approach used by McVean [89] to provide a genealogical interpretation of principal components analysis in genetics.

In a related approach, several recent methods in statistical genetics [58,59,90] and in phylogenetics [91] take as input a genome-wide inference of local gene trees. If the gene trees are treated as known, then the only source of randomness is the placement of mutations, as in equation S7, and averaging over trees is accomplished by taking an average over the estimated gene trees. For example, Link and colleagues [58] compute the expectation of a local GRM (i.e., a local eGRM) conditional on estimated gene trees in a region of the genome. These local eGRMs are then used as input to a variance-components model, which brings some advantages in mapping QTLs. Specifically, the resulting (conditional) expected genetic relatedness matrices naturally incorporate LD, providing better estimates of local genetic relatedness than could be formed from a handful of SNPs in a local region [58,90].

#### 2.2.4. The phylogenetic variance-covariance matrix

In an extreme case, we might consider only variation among long-separated species. If we ignore incomplete lineage sorting, there may be only a single tree that describes the relationships among species, and the expectation over gene trees used in the previous subsection can be dropped, leaving us with equation S7. Then the entries of the relatedness matrix Σ, which in the case of phylogenetic methods is referred to as the phylogenetic variance-covariance (or vcv), are given by

$$Cov(A_{i},A_{j})=\frac{LE(\beta^{2})}{T}T_{ij}.$$ (12)

This can be recognized as the covariance under the Brownian motion model [45] commonly used to model continuous traits in phylogenetics, given a phylogenetic tree, when setting the diffusion rate *σ*^2^ of the Brownian motion process to

$$\sigma^{2}=\frac{LE(\beta^{2})}{T}.$$ (13)

Eq 13 may look unlike expressions for *σ*^2^ in phylogenetics, where the Brownian motion rate is typically taken to be *V*_*A*_/*N*, where *V*_*A*_ is the additive genetic variance and *N* is the effective population size, following Lande [92], or simply $UE(\beta^{2})$, where *U* represents the total mutation rate toward causative alleles, following Lynch and Hill [93]. To reconcile our result with the existing literature, note that if mutations occur on the tree as a Poisson process with a rate *U* per unit of tree length, then E(L)=UT, so that

$$E(\sigma^{2})=\frac{E(L)E(\beta^{2})}{T}$$

$$=UE(\beta^{2}),$$

as shown by Lynch and Hill [93]. Further, under a neutral model, the equilibrium additive genetic variance *V*_*A*_ is proportional to $NUE(\beta^{2})$ [94]. Thus, under neutrality,

$$\frac{V_{A}}{N}\propto \frac{NUE(\beta^{2})}{N}$$

$$=UE(\beta^{2}),$$

showing that under a neutral model, the Lande formulation is equivalent to the Lynch and Hill formulation, up to constants that depend on ploidy. Thus, we see that our Eq 13 matches familiar formulations in the literature [95].

Consistent with previous arguments (e.g., [35]), this result also implies that one interpretation of the standard PGLS model is that it stratifies the regression between focal variables by an unobserved variable (or variables) that evolved primarily by drift. Hansen and colleagues have pointed out that this may not be an appropriate model for testing for adaptation [32,33,96], which was the primary motivation for developing many comparative methods in the first place [97]. Moreover, recently, standard PGLS has come into question in scenarios in which there is discordance between the gene tree and the species tree [98–100]. Our formulation makes it clear that the standard PGLS formulation only applies when there is a single tree underlying all loci; if there is instead a distribution of gene trees, equation S8 suggests that the appropriate thing to do is to average over gene trees, as suggested by Hibbins and colleagues [99], and as done in a statistical genetics setting [58,59]. However, one difficulty is deciding over which gene trees one should average, particularly if the trait is oligogenic [100].

#### 2.2.5. Connections among different approaches to modeling genetic contributions to phenotypic covariance

**Fig 1:**
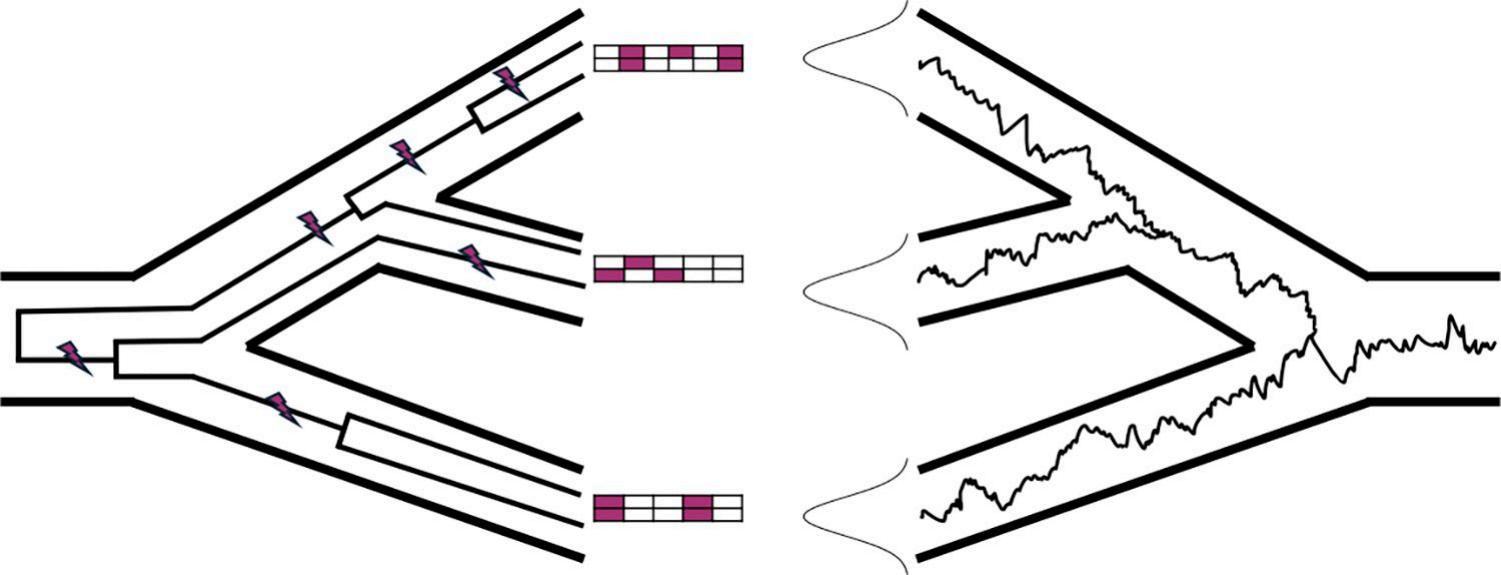
Relationship between different models of phenotypic covariance. The left-hand side shows the situation when multiple samples are taken from each group, as is the case in a genome-wide association study. The population tree is indicated by bold lines, and inside of it gene trees are indicated by thinner lines. Mutations on the gene trees are indicated by purple lightning bolts. The mutations on the gene tree result in genotype matrices, shown as one 2 × 5 array per species, with purple-filled entries indicating mutations. The right-hand side shows the situation in phylogenetics, where the species mean phenotype, indicated by a thin squiggly line, evolves according to a Brownian motion within a species tree, indicated by bold lines. The distribution of possible phenotypes within each species is marginally Gaussian.

Fig 1 provides a conceptual picture of how the various approaches are related to each other. The left side shows the situation typical in genome-wide association settings: SNP genotypes, shown as a matrix of variable sites with derived alleles colored in red, are determined by the topologies of gene trees and the mutations that fall on them. The GRM is computed on the basis of the SNP genotypes, as in Eq 12. If gene trees are known, then the eGRM can be computed by averaging over Poisson placement of mutations as in equation S7 over gene trees. If only a demography is known, both gene trees and mutations can be averaged over using coalescent theory, as in equation S8. The right-hand side shows the situation in phylogenetics: on a single fixed tree, the population trait mean evolves according to a Brownian motion. This results in a multivariate Gaussian distribution of phenotypes across species. We show that the covariance predicted by the Brownian motion model is equivalent to the covariance predicted by averaging over Poisson distributed mutations on a gene tree that is fixed to coincide with the species tree. In the figure, we highlight bifurcating population trees for simplicity and clarity, but the results also apply in complex demographic scenarios with admixture and reticulation.

### 2.3. How the same type of unmodeled structure misleads both GWAS and phylogenetic regressions

That standard models in statistical genetics and phylogenetics are deeply related immediately suggests that these models might suffer the same pathologies under model misspecification, and that solutions to these pathologies could be shared across domains. Here, we illustrate this by studying the problem of how unmodeled (phylo)genetic structure biases estimates of regression covariates. This problem has received much attention in both the statistical genetics [101,102] and phylogenetics literature [34,35,103], but the approaches taken in the 2 fields differ.

We assume that we have a sample of size *n* with a predictor, *x* = (*x*_1_, *x*_2_,…,*x*_*n*_)^*T*^, and a trait, *y* = (*y*_1_, *y*_2_,…,*y*_*n*_)^*T*^. In the context of GWAS, *x* may be the (centered) genotypes at a locus to be tested for association, while in the context of phylogenetics, *x* is often an environmental variable or another trait that is hypothesized to influence *y*. Then, the regression model is

$$y_{i}=x_{i}\beta +A_{i}+E_{i},$$ (14)

where *A*_*i*_ and *E*_*i*_ are the genetic and environmental components, as in Eq 2, and *β* is the effect of *x* on *y*. In genome-wide association studies, *β* is the effect size of the locus being examined, while in phylogenetics it may quantify the effect of an environmental variable or other continuous trait, rather than the effect of an allele. *A* is not generally known and so cannot be incorporated in the regression directly, raising the possibility that apparent effects of *x* may in fact be due to *A*, if *A* and *x* are correlated. Even though *A* is unknown, if we know how individual values of *A* covary, then we can correct for that covariance rather than correcting for *A* itself.

#### 2.3.1. Theoretical analysis

To understand the purpose and limitations of corrections for (phylo)genetic structure, we examined the properties of the estimators of regression coefficients with and without correction for (phylo)genetic structure. To do so, we diagonalize the genetic covariance matrix, Σ = *V*Λ*V*^*T*^ where $V=[\begin{array}{llll} v_{1} & v_{2} & \cdots & v_{n} \end{array}]$ is a matrix whose columns are the eigenvectors of Σ, and Λ = diag(*λ*_1_, *λ*_2_,…,*λ*_*n*_) is a diagonal matrix whose diagonal contains the eigenvalues of Σ. Σ, by virtue of being a covariance matrix, is guaranteed to be positive semidefinite. Thus, by the spectral theorem, the eigenvectors of Σ can be used to form an orthonormal basis of ℝ^*n*^. In practice, Σ may have repeated eigenvalues, and hence the eigenvectors may need to be orthogonalized; intuitively, these repeated eigenvalues correspond to individuals, populations, or species that share the same evolutionary history. We proceed by assuming that the eigenvectors of Σ have been orthogonalized.

The simplest estimator of the relationship between 2 variables is the ordinary least squares estimator,

$${\hat{\beta }}^{\left(OLS\right)}=\frac{\sum_{j=1}^{n}(v_{j}^{T}x)(v_{j}^{T}y)}{\sum_{j=1}^{n}(v_{j}^{T}x)(v_{j}^{T}x)}.$$ (15)

This shows that we can conceptualize the ordinary least squares estimator as adding up the correlations between *x* and *y* projected onto each eigenvector of Σ. Loosely, large-magnitude slope estimates arise when *x* and *y* both project with large magnitude onto one or more eigenvectors of Σ. If an eigenvector of Σ is correlated with a confounding variable, such as the underlying (phylo)genetic structure, then *x* and *y* may both have substantial projections onto it, even if *x* and *y* are only spuriously associated due to the confound.

Two seemingly distinct approaches have been proposed to address this issue. First, researchers have proposed including the eigenvectors of Σ as covariates. In the phylogenetic setting, this is known as phylogenetic eigenvector regression [104]. (In practice, researchers often use the eigenvectors of a distance matrix derived from the phylogenetic tree rather than Σ itself, but these 2 matrices have a straightforward mathematical connection [105].) In the statistical genetics setting, the analogous approach is to include the principal component projections of the data that are used to generate the genetic relatedness matrix—i.e., the principal components of the genotype matrix [23]—in the regression. For completeness, in Section D in S1 Text we show that these 2 approaches include the same covariates, up to a scaling factor.

In Section E in S1 Text, we show that, when the first *J* eigenvectors of Σ are included as covariates, the estimate of the coefficient of the predictor *x* is

$${\hat{\beta }}_{x}^{\left(OLS,EIG\right)}=\frac{\sum_{j=J+1}^{n}(v_{j}^{T}x)(v_{j}^{T}y)}{\sum_{j=J+1}^{n}(v_{j}^{T}x)(v_{j}^{T}x)}.$$ (16)

This is straightforwardly the OLS estimator (Eq 15), except that the first *J* eigenvectors of Σ are removed. This shows why inclusion of the eigenvectors of Σ as covariates can correct for (phylo)genetic structure: it simply eliminates some of the dimensions on which *x* and *y* may covary spuriously. However, it also shows the limitations of including eigenvectors as covariates. First, because it is simply cutting out entire dimensions, it can result in a loss of power. Second, confounding that aligns with eigenvectors that are not included in the design matrix is not corrected.

The second approach to including the eigenvectors of Σ as covariates is to use Σ itself to model the residual correlation structure. In phylogenetic biology, this is accomplished using phylogenetic generalized least squares (PGLS) [39,40], whereas in statistical genetics this is accomplished using LMMs [28,106]. (We work with generalized least squares below; for a similar argument in an LMM setting, see [27].) In both settings, it is common to add a “white noise” or “environmental noise” term, such that the residual covariance structure is $\sigma_{G}^{2}\Sigma +\sigma_{E}^{2}I$, where $\sigma_{G}^{2}$ scales the contribution of genetics, $\sigma_{E}^{2}$ scales the contribution of environment, and *I* is the identity matrix. In the context of phylogenetics, the relative sizes of $\sigma_{G}^{2}$ and $\sigma_{E}^{2}$ are of interest when estimating the phylogenetic signal measurement Pagel’s lambda [107,108], whereas in statistical genetics, they are the subject of heritability estimation [109]. Then, both PGLS and LMM approaches model the data as follows:

$$y∼Multivariate\;Normal(\beta x,\sigma_{G}^{2}\Sigma +\sigma_{E}^{2}I),$$

where $\sigma_{G}^{2}$ and $\sigma_{E}^{2}$ are typically estimated, for example, by maximum likelihood [110], residual maximum likelihood [74], Haseman–Elston regression [111,112], or other methods [28,106,113]; see Min and colleagues [114] for a comparison some estimation approaches and an examination of the impact of linkage disequilibrium. For the theoretical analysis that follows, we assume $\sigma_{G}^{2}=1$ and $\sigma_{E}^{2}=0$. This does not restrict the applicability of our analysis, because $\sigma_{G}^{2}\Sigma +\sigma_{E}^{2}I$ has the same eigenvectors as Σ, with corresponding eigenvalues $\sigma_{G}^{2}\lambda_{i}+\sigma_{E}^{2}$, where *λ*_*i*_ are the eigenvalues of Σ.

In Section F in S1 Text, we show that the GLS estimate of the regression coefficient is

$${\hat{\beta }}_{x}^{\left(GLS\right)}=\frac{\sum_{j=1}^{n}\lambda_{j}^{-1}(v_{j}^{T}x)(v_{j}^{T}y)}{\sum_{j=1}^{n}\lambda_{j}^{-1}(v_{j}^{T}x)(v_{j}^{T}x)}.$$ (17)

Like the ordinary least squares estimator in Eq 18, this expression includes all the eigenvectors of Σ. However, it downweights each eigenvector according to its eigenvalue. Thus, GLS downweights dimensions according to their importance in Σ, which aims to describe the structure according to which *x* and *y* may be spuriously correlated. However, unlike Eq 16, it retains all dimensions. Compared with adjusting for the leading eigenvectors of Σ using OLS, the GLS approach retains some ability to detect contributions to associations that align with the leading eigenvectors. It also adjusts for Σ in its entirety, rather than just its leading eigenvectors. This means that it adjusts for even very recent (phylo)genetic structure, which will likely not be encoded by the leading eigenvectors. That said, one disadvantage of GLS is that it assumes that all eigenvectors of Σ contribute to confounding in proportion to their eigenvalues, potentially resulting in an inability to completely control for confounding if the effect of an eigenvector of Σ is not proportional to its eigenvalue, as may be the case with, for example, environmental confounding. In other words, the cost of including some adjustment for every eigenvector of Σ is an assumption as to how these eigenvectors relate to confounding.

Where sample sizes and computational resources allow it, typical recent practice in statistical genetics is to use a linear mixed model framework while also including some eigenvectors of Σ as covariates [28,106,113]. This at first may seem surprising, because it seems to be controlling for Σ twice. However, the analysis above suggests that including the eigenvectors as covariates and using GLS have different, and perhaps complementary, effects on the resulting estimates. To see how they interact, we show in Section F in S1 Text that the estimate of the regression coefficient of *x*,

$${\hat{\beta }}_{x}^{\left(GLS,EIG\right)}=\frac{\sum_{j=J+1}^{n}\lambda_{j}^{-1}(v_{j}^{T}x)(v_{j}^{T}y)}{\sum_{j=J+1}^{n}\lambda_{j}^{-1}(v_{j}^{T}x)(v_{j}^{T}x)}.$$ (18)

Thus, using the eigenvectors of Σ as covariates in a generalized least squares framework may provide the benefits of both approaches: if there is confounding in a eigenvector of Σ that is “too large”—that is, it is out of proportion with its associated eigenvalue—then if that eigenvector is included in the design matrix, it will simply be excised from the estimator, as in Eq 17. However, we still maintain the ability to control for spurious association between *x* and *y* due to the structure of Σ but not along included eigenvectors, as in Eq 17. The major difficulty is in identifying the eigenvectors of Σ that might be associated with confounding effects larger than their corresponding eigenvalues would suggest.

#### 2.3.2. Simulation analysis

To put the intution developed from the previous subsection into practice, we performed simulations in both phylogenetic and statistical-genetic settings. First, to explore how the approaches outlined above correct for both (phylo)genetic structure and environmental confounding, we performed simulations inspired by Felsenstein’s “worst case” scenario [35,46]. Felsenstein’s worst case supposes that there are 2 diverged groups of samples that are measured for 2 variables *x* and *y*, which are then tested for association; the only (phylo)genetic structure is between the 2 groups. In the phylogenetic setting, we represent the 2 clades as star trees with 100 tips each, connected by internal branches, and we simulate *x* and *y* as arising from independent instances of Brownian motion along the tree (see Methods). In the statistical genetics setting, we use msprime [115] to simulate 100 diploid samples from each of 2 populations, and then simulated quantitative traits using the alpha model [22] (see Methods). In this setting, McVean [89] showed that the first eigenvector of Σ captures population membership; hence, we only include the first eigenvector to capture any residual confounding. To perform inference in the phylogenetic case, we used the package phylolm [110], and for the statistical-genetic case, we used a custom implementation of REML [74].

**Fig 2:**
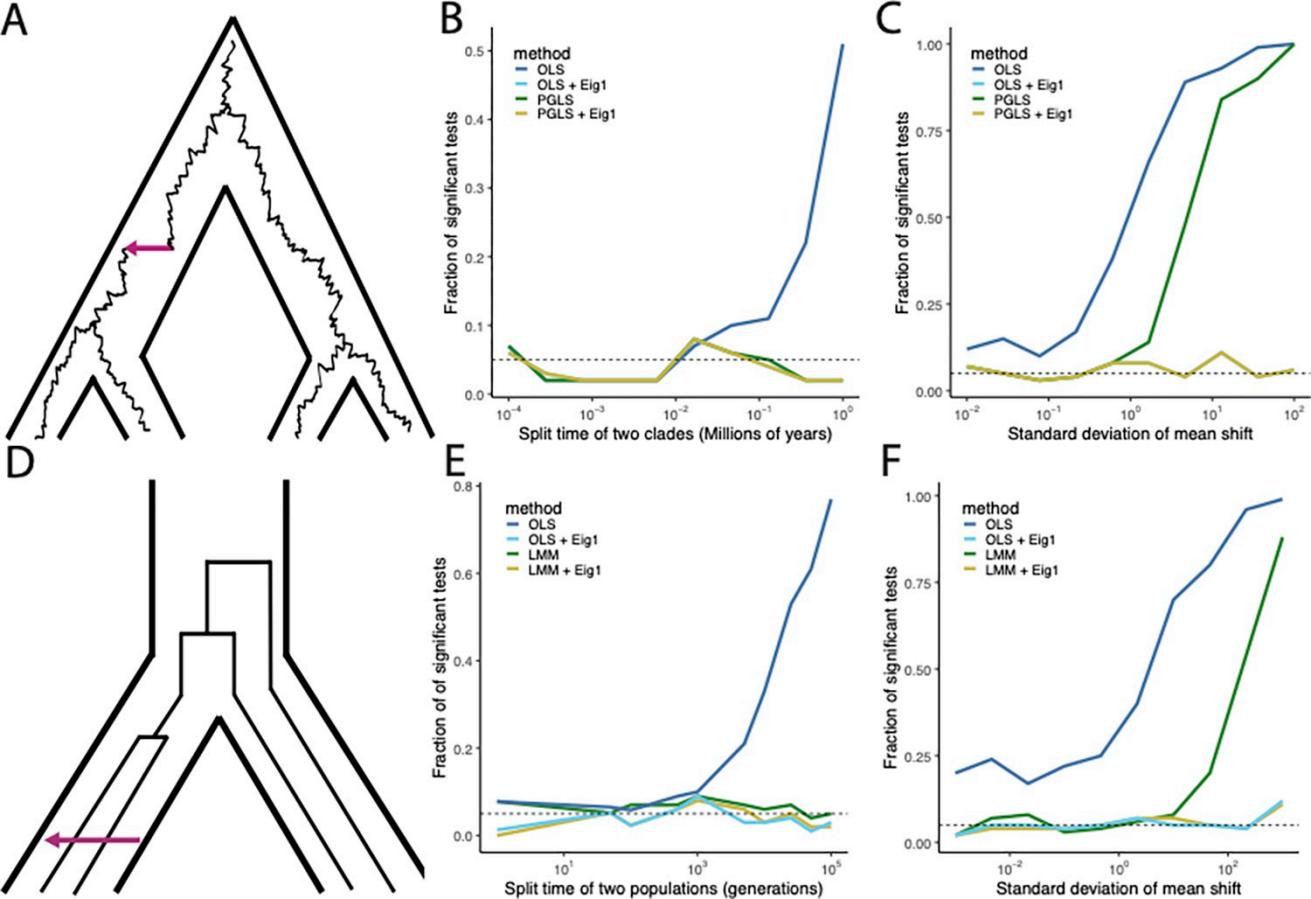
Performance of different methods for controlling confounding in Felsenstein’s worst case. (A) A depiction of Felsenstein’s worst case in the phylogenetic setting. A Brownian motion evolves within a species tree separating 2 clades. For simplicity, 2 tips are shown in each clade; in the simulations, each clade contains 100 tips. The purple arrow shows a simulated singular evolutionary event (see text). (B) The false positive rate of each method in a simulated phylogenetic regression as a function of divergence time between the 2 groups. The horizontal axis shows the divergence time, while the vertical axis shows the fraction of tests that would be significant at the 0.05 level. Each line represents a different method. The lines for OLS + Eig1 and PGLS + Eig1 are completely overlapping. (C) The false positive rate of each method in a simulated phylogenetic regression as a function of the size of non-Brownian shifts in both predictor and response variables. The horizontal axis shows the standard deviation of the normal distribution from which the shift was drawn, and the vertical axis shows the fraction of tests that would be significant at the 0.05 level. The lines for OLS + Eig1 and PGLS + Eig1 are completely overlapping. (D) A depiction of Felsenstein’s worst case in the statistical genetic setting. Gene trees with mutations are embedded within a population tree depicting 2 divergent populations. For simplicity, 2 samples are shown within each population; in the simulations, each population consists of 100 diploid individuals. The purple arrow shows a simulated environmental effect (see text). (E) The false positive rate of each method in a simulated GWAS as a function of divergence time between the 2 groups. The horizontal axis shows the divergence time, while the vertical axis shows the fraction of tests that would be significant at the 0.05 level. Each line represents a different method. (F) The false positive rate of each method in a simulated GWAS as a function of the size of an environmental shift. The horizontal axis shows the standard deviation of the normal distribution from which the shift was drawn, and the vertical axis shows the fraction of tests that would be significant at the 0.05 level. Underlying data can be found at https://zenodo.org/records/13774370.

We first explored the impact of deepening the divergence between the 2 clades, starting from no divergence and increasing to high divergence (Fig 2A and 2C). As expected, we see ordinary least squares fails to control for the population stratification as the divergence time becomes large, resulting in excessive false positives. However, all of the other approaches appropriately controlled for the population stratification. This is as expected: in the case of 2 populations, all of the (phylo)genetic stratification is due to the accumulation of genetic variants in each group. Hence, either discarding the correlation between *x* and *y* on the dimension corresponding to group membership as in Eq 16 or downweighting it as in Eq 18 is sufficient to remove the confounding effect of the population stratification.

Despite the success of both OLS with eigenvector covariates and generalized least squares in controlling for population stratification, it has recently been recognized that phylogenetic generalized least squares does not control for all types of confounding in Felsenstein’s worst case: for example, if there is a large shift in *x* and *y* on the branch leading to one of the groups, phylogenetic generalized least squares produces high false positive rates [35]. Because including the first eigenvector of Σ will completely eliminate the contribution to the estimated coefficient that projects on group membership, whereas generalized least squares will only downweight it, we reasoned that including the first eigenvector in either ordinary or generalized least squares should restore control even in the presence of large shifts.

We tested our hypothesis using simulations with divergence time in which ordinary least squares was not sufficient to correct for population stratification. In the phylogenetic case, we simulated an additional shift in one of the clades for both *x* and *y* by sampling from independent normal distributions, while in the statistical-genetic case, we simulated an environmental shift sampled from a normal distribution in one of the clades (Fig 2B and 2D). As expected, ordinary least squares is insufficient to address the confounding, and becomes increasingly prone to false positives as the size of the shift increases. In line with our hypothesis, phylogenetic generalized least squares and linear mixed modeling also fail to control for the shift as it becomes large, while including just a single eigenvector in each case is sufficient to regain control over false positives.

The preceding analysis might suggest that including eigenvectors of Σ as covariates is sufficient to adjust for (phylo)genetic structure while also being superior to generalized least squares in dealing with environmental confounding. Recent work, however, suggests that inclusion of principal components may not be able to adjust for more subtle signatures of population structure [8,15,102,116]. To explore this, we simulated both phylogenetic regression and a variant association test using a more complicated model of population structure. For the phylogenetic case, we simulated pure birth trees with 200 tips, while in the statistical genetics case, we simulated pure birth trees with 20 tips and sampled 10 diploids from each tip using msprime. Then, as before, we simulated using a Brownian motion model in the phylogenetic case, or an additive model for the statistical genetic case.

**Fig 3:**
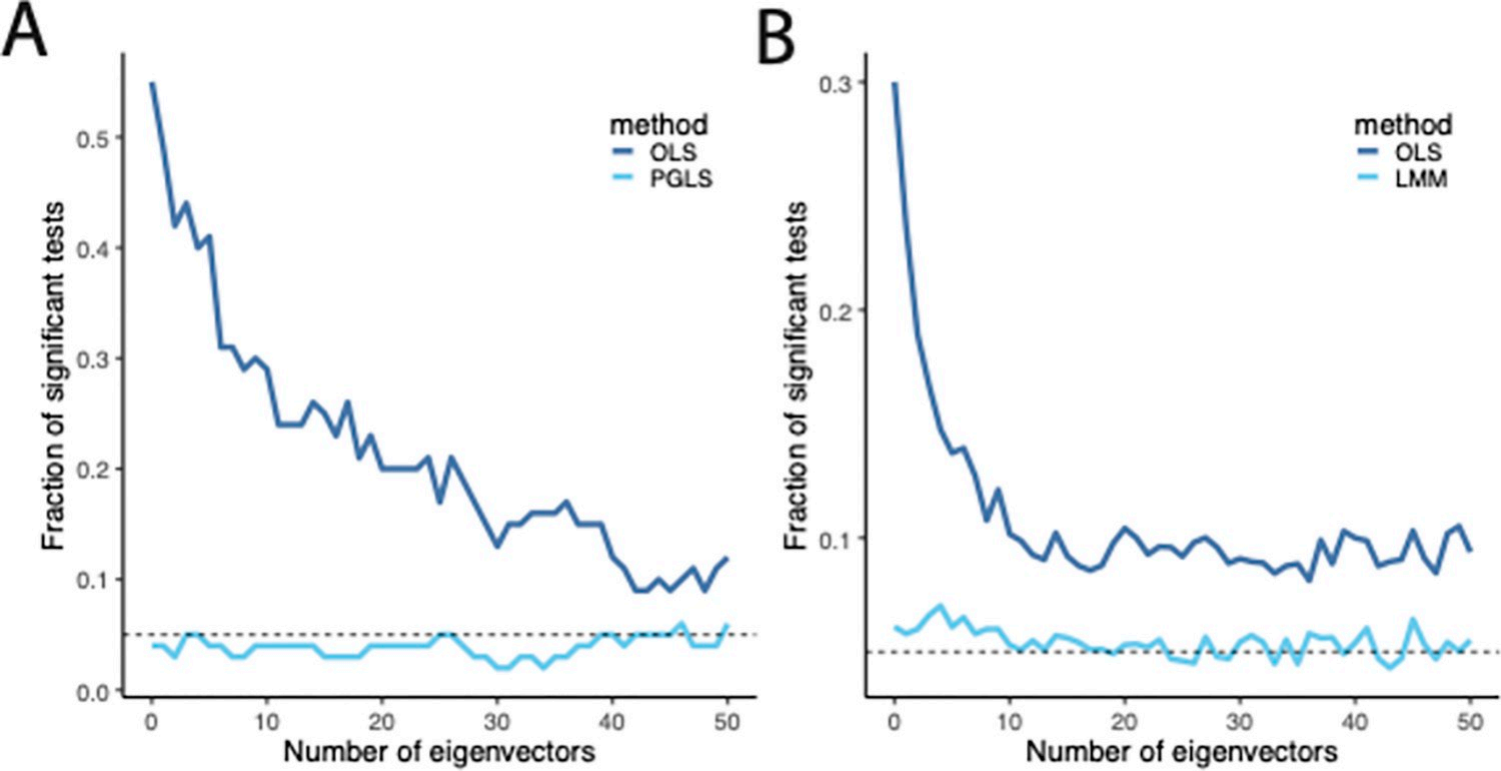
The performance of eigenvectors of the covariance matrix in a model with more complex population structure. (A) Performance of ordinary least squares and phylogenetic least squares in a model with 200 tips related by a pure birth tree. The horizontal axis shows the number of eigenvectors included as covariates, and the vertical axis shows the fraction of tests that would be significant at the 0.05 level. (B) Performance of ordinary least squares and a linear mixed model in a model with 20 populations related by a pure birth tree and 10 diploid individuals per population. The horizontal axis shows the number of eigenvectors included as covariates, and the vertical axis shows the fraction of tests that would be significant at the 0.05 level. Underlying data can be found at https://zenodo.org/records/13774370.

As expected, using ordinary least squares without any eigenvector covariates does not control for population structure in either the phylogenetic or the statistical-genetic setting, but the methods that use generalized least squares estimates of the regression coefficients appropriately model population structure (Fig 3). Although adding additional eigenvectors reduces the false positive rate of ordinary least squares, false positives are not reduced to the nominal level of 5%. This is in line with our theoretical analysis: as seen in Eq 16, including eigenvectors in ordinary least squares eliminates dimensions that explain the most genetic differentiation, but the correlations on the remaining dimensions are not adjusted. Because there is substantial fine-scale population structure in these simulations, removal of just a few dimensions with large eigenvalues is not sufficient to control for the subtle signature of population structure. In the phylogenetic setting, we expect that including additional eigenvectors would eventually gain control of false positives, but it may require including all of the eigenvectors and result in an overdetermined problem. On the other hand, in the population-genetic simulations, including additional eigenvectors will not increase control over false discoveries. There are 2 reasons for this. First, because Σ is estimated from the genetic data, the eigenvectors themselves are estimated. In practice, this means that eigenvectors corresponding to small eigenvalues are estimated poorly. Second, because we have 200 samples but only 20 populations, many of the samples share the same evolutionary history, and hence several eigenvectors share the same eigenvalue “in theory”—that is, if viewed from the perspective of the population tree rather than the realized gene trees or genotypes. Roughly speaking, in this simulation, there are only approximately 20 eigenvectors that correspond to “true” confounding. In practice, due to randomness of mutations and gene trees, the remaining eigenvectors will not share identical eigenvalues, but will nonetheless correspond to genetic differentiation of individuals with shared evolutionary history, and hence will not correct for genetic confounding. This is reminiscent of the observations that in some human genetics data sets, only the first few eigenvectors stably capture genetic differentiation [5], and that LMM approaches become increasingly necessary when the sample includes relatively close genealogical relatives, whose relatedness is captured in the GRM but will not typically affect its leading eigenvectors [102].

In contrast to including eigenvectors as fixed effects as part of an OLS analysis, generalized least squares approaches, as shown in Eq 18, will continue to correct for population structure that is found deeper into the eigenvectors of the correlation matrix (echoing points previously raised in the phylogenetics literature [117–119]). We also note that while the our analysis is focused on the eigenvectors of Σ, we suspect similar lines of reasoning may apply to other situations in which eigenvector regression is used, such as in spatial ecology [120].

### 2.4. A case study of including eigenvectors as covariates in PGLS

Although the eigenvectors of the phylogenetic variance-covariance matrix (or closely related quantities) have often been included in regression models by researchers using phylogenetic eigenvector regression [104], to the best of our knowledge, phylogenetic biologists have not previously used these eigenvectors as fixed effects in a PGLS model, which we have shown above to be a potentially effective strategy in theory. To illustrate the approach in practice, we re-examine a recent study by Cope and colleagues [121] that tested for coevolution in mRNA expression counts across 18 fungal species. More specifically, these researchers were interested in testing whether genes whose protein products physically interacted (using independent data from [122]) were more likely to have correlated expression counts than those whose protein products did not. They found support for this prediction. While we suspect the core finding is robust, and there are some theoretical reasons to expect that RNA expression counts should be Brownian-like under some selective scenarios [123], other studies have shown expression counts for many genes in this data set (and many others) are not well described by a Brownian process [124,125]. As such, some of their observed correlations could be spurious due to unmodeled phylogenetic structure [35].

**Fig 4:**
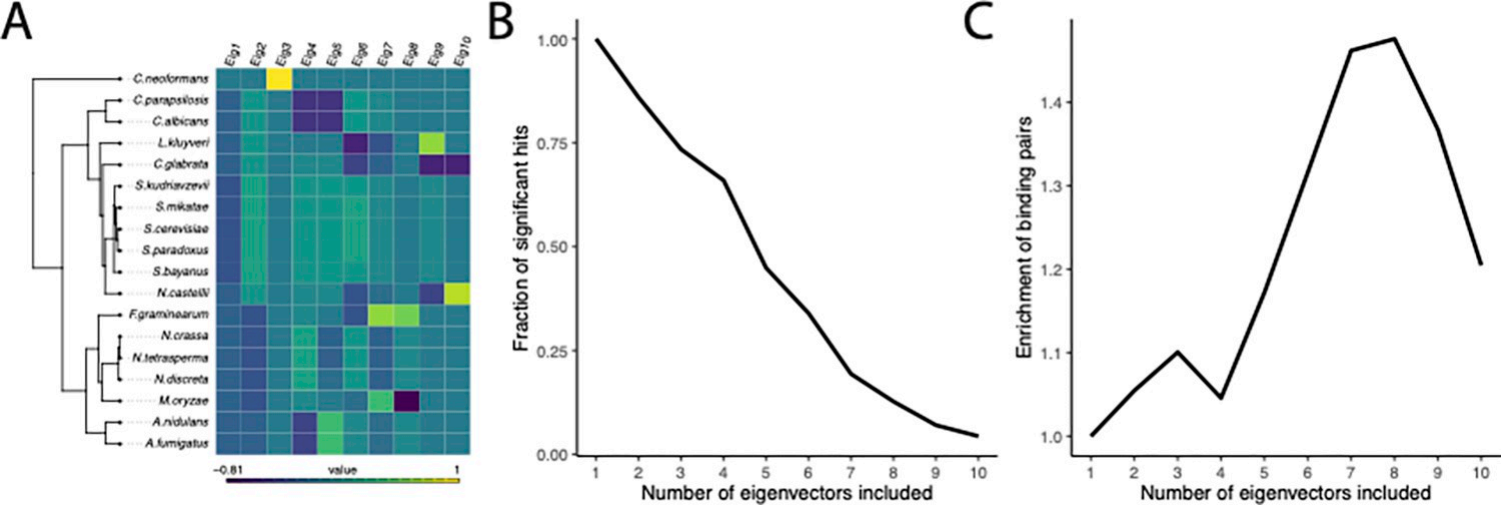
Impact of including phylogenetic eigenvectors on detection of coevolution of gene-expression levels in fungi. (A) The fungal tree; colors indicate each species’ position in the first 10 dimensions of principal component space. (B) The overall number of significant pairs decreases as more eigenvectors are included in the regression. The horizontal axis indicates the number of eigenvectors included as fixed effects, and the vertical axis shows the proportion of significant pairs compared with a model that includes no eigenvectors as fixed effects. (C) The enrichment of known binding pairs as a function of eigenvectors included. The horizontal axis indicates the number of eigenvectors included as fixed effects, and the vertical axis shows the enrichment of known binding pairs relative to a model in which no eigenvectors are included. Underlying data can be found at https://zenodo.org/records/13774370.

We re-analyzed the data of Cope and colleagues [121] with the addition of the eigenvectors of (phylogenetic) Σ as fixed effects in the PGLS model (see Methods and materials for details). Cope and colleagues used a correlated multivariate Brownian model to test their hypothesis, which is slightly different from the more common PGLS approach [126], but they are close enough for our purposes. We conducted several iterations of the analyses, varying the number of eigenvectors included from 1 to 10; Fig 4A shows how the different species project onto each principal component. We found that, as anticipated, the number of significant correlations decreased as more eigenvectors were included (Fig 4B). However, as more eigenvectors were included, the proportion of significant correlations in gene-expression count data in which the genes are known to physically interact increased (up to about 8 eigenvectors; Fig 4C). If we assume that the significant correlations for physically interacting genes are more likely to be true positives than those for pairs of genes not known to interact physically, then the results would suggest that including the eigenvectors in the analysis might reduce the false positive rate while still finding many of the true positives.

Uyeda and colleagues [35] suggest that one way to mitigate the spurious correlations arising from large, unreplicated events would be to include indicator variables in the regression model that encode the part of the phylogeny from which a tip descends. This is similar in spirit to the use of hidden Markov models for the evolution of discrete traits [103,127]. However, as Uyeda and colleagues point out, this leaves open the hard problem of identifying the branches on which to stratify. It is not possible to include an indicator for every branch, as the model would then be overdetermined. Using the simple method borrowed from GWAS studies of including eigenvectors of Σ as fixed effects in the typical phylogenetic regression may be a promising (partial) solution to the problem of spurious correlations.

## 3. Discussion

### 3.1. The genetic model versus the statistical model

We began by adding assumptions to a general model of a polygenic trait (Eq 2) in order to show that common practices in disparate areas of genetics can be seen as special cases of the same model. One notable assumption is that of a purely additive model [128] for the phenotype (Eq 1). There are 2 reasons we might be suspicious of this assumption. First, it is debatable to what extent most traits obey the additive model, given evidence of non-additive genetic contributions to traits across species [129,130]. However, even if non-additive contributions are important for determining individual phenotypes or for understanding traits’ biology, they might still contribute a relatively small fraction of trait variance, meaning they might be safely ignored for some purposes [131–133] (but see [134]). Second, we used a neutral coalescent model to find an expression for the Brownian motion diffusion parameter in terms of the effect sizes of individual loci (Eq 13). Although this provides a satisfying justification for the use of a phylogenetic regression model with a Brownian covariance structure and for averaging over gene trees to accommodate ILS (*sensu* [99]), it is likely unreasonable in many situations. It has long been appreciated that, while a population-mean phenotype will be expected to evolve according to a Brownian process under simple quantitative-genetic models of genetic drift [43,92,95,135] the Brownian rate estimated from phylogenetic comparative data is orders of magnitude too slow to be consistent with plausible values for the quantitative-genetic parameters used to derive the Brownian model [95,135–137]. There are more elaborate explanations than pure genetic drift for why long-term evolution may show relatively simple dynamics [138] but understanding the coalescent patterns of loci under these scenarios is likely challenging [139] and beyond the scope of the present paper.

However, even if one finds the genetic model unreasonable, the equivalence of the *statistical* models used in statistical genetics and phylogenetics still holds: that is, the core structures of the models are the same, whether one is willing to interpret the parameters in the same way or not. Indeed, phylogenetic biologists have been here before, with the realization that PGLMMs are structurally equivalent to the pedigree-based analyses using the animal model from quantitative genetics [40–42] even though the recognition that they were equivalent did not rely on a specific genetic model for phenotypes. (We showed here that they can both be derived from the same genetic model.) Nonetheless, the recognition of a structural equivalence between the animal model and the phylogenetic model made it possible to use techniques from quantitative genetics to solve problems in phylogenetic comparative methods. For example, inspired by a similar model from [140], Felsenstein developed a phylogenetic threshold model [141,142], in which discrete phenotypes are determined by a continuous liability that itself evolves according to a Brownian process. Hadfield [143] proved this model was identical to a variant of the animal model and that existing MCMC algorithms could be used to efficiently estimate parameters and extend the threshold to the multivariate case, which had not been previously derived.

### 3.2. Towards a more integrative study of the genetic bases of phenotypes

Building a general framework is a step towards inference methods that coherently integrate intra- and interspecific variation to understand the genotype-to-phenotype map and how evolutionary processes, acting at different time scales, shape it. Indeed, the importance of evolutionary conservation in triaging functional variants in the human genome has long been appreciated and is becoming increasingly important as we collect larger samples of people; the same is true for the use of genomics in agriculture [57] and conservation genetics [55]. Recent work showed that evolutionary conservation accounts for the vast majority of the predictive power of a state-of-the-art deep learning approach to variant annotation [144,145]. But most of the cutting-edge phylogenomic approaches for triaging variants typically do not use the phylogeny at all (i.e., only multiple sequence alignments [MSAs] are used), or include the phylogeny without an explicit evolutionary model [146]. This is a limitation because we are not making the most of the information in the tree, nor are we able to draw specific inferences about how evolutionary processes have shaped complex traits from the MSA alone. Overcoming this limitation is not straightforward and will require mechanistic modeling: The observed level of conservation is a nonlinear function of the strength of selection acting against variants at a locus; small changes in the strength of negative selection can greatly decrease the amount of variability seen on phylogenetic timescales, and this can cause counterintuitive behavior of conservation scores [54,147].

A key difficulty in combining information across timescales arises from different assumptions about the evolutionary process. For example, the canonical GRM in statistical genetics assumes that the variance of an allele’s effect size is inversely proportional to the heterozygosity at the locus. As we show in Section A in S1 Text, this assumption can be justified under a model of mutation-selection balance with Gaussian stabilizing selection on a single trait. However, we do not generally understand how robust such approaches are under more complex (and realistic) evolutionary scenarios that include the influence of genetic drift and selection on genetically correlated traits, nor how errors influence downstream inferences [71,76,148,149]. There is substantial evidence that rarer variants tend to have larger effect sizes [76,150–155], which is broadly consistent with the motivation for the canonical GRM and for the more general *α* model, which supposes that the variance of the effect size of an allele is given as a power law function of its heterozygosity [22,68,74,76]. (Although we show here that setting *α* = 1 can be motivated by a model of stabilizing selection on a single trait and ignoring genetic drift, the more general *α* model is not derived from an evolutionary model.) However, close examination of GWAS effect sizes suggests a poor fit of the *α* model for many traits [148], and it has been suggested that more complex models might better capture the wide variation of effect sizes [155]. Further, recent explosive human population growth has resulted in a massive number of rare variants [156–160]—assuming that there is substantial input of selectively neutral mutations, some of these rare variants will be rare not because they have been driven to or held at low frequency by selection, but simply because they represent the effect of population growth on the neutral site-frequency spectrum. As such, using the alpha model may result in overestimation of heritability for traits where there is a substantial contribution of genetic drift and may result in incompletely controlled confounding in trait mapping studies. And although effect sizes of individual causal variants can be estimated well for common variants, this is unlikely ever to be possible for sufficiently rare variants; hence, a realistic model of effect sizes as a function of allele frequency is necessary for inclusion in efforts such as rare-variant association studies [52,161–163].

In contrast, in our derivation of gene-tree (i.e., those using the eGRM) and phylogenetic (i.e., using the phylogenetic variance-covariance matrix) model, we assumed that effect sizes and genotypes were independent, and that trait-affecting mutations fall on gene trees as a Poisson process [89]. These assumptions are justified if the causal variants are neutral. But the neutrality assumption contradicts a wealth of evidence from both within and among species that quantitative trait variation is under some form of selection [164–171] and that the effect sizes of causal variants tend to be larger in more evolutionarily conserved regions [50,144,172–175], which also implies an important role of purifying selection. The *α* model, or presumably other models of the relationship between effect size and allele frequency, can be incorporated in an eGRM [59,90]. After all, an eGRM is an expectation (under Poisson-process mutation) of a GRM, and so any scaling applied to genotypes in computing the GRM can be made to apply to the eGRM. But the interpretation becomes complicated, since the assumption that mutations accrue on the tree as a Poisson process is still being relied upon.

One way phylogenetic biologists include selection is by modeling the evolution of quantitative traits with an Ornstein–Uhlenbeck (OU) process [96,176–179], which can be derived from a quantitative-genetic model of stabilizing selection [92], although in practice, the OU model is often interpreted as a phenomenological model of the evolution of the adaptive peaks [44,180]. Many researchers have used the Σ matrix derived from an OU process in PGLS models [176,181]; this is straightforward because the data remain multivariate Gaussian [39,110]. One could potentially use an analogous approach to model phenotypic evolution along gene trees within a species (to inform the construction of eGRM, for example). Such an approach could improve inferences from both tree-based GWAS (sensu [58,59]) and from emerging phylogenetic comparative approaches that consider gene trees rather than just the species trees [98,99,182] (such approaches are important as only using a single species tree may lead one to mistake similarity due to common ancestry for convergence [100,183–185]). However, identifying the correct form of the model would likely require an analysis of the ancestral selection graph [139,186], a notoriously challenging theoretical endeavor.

In sum, an implication of our results is that standard approaches in both statistical genetics and phylogenetic comparative methods incorporate assumptions that are plausibly motivated under neutrality but questionable under various forms of selection—ignoring covariances among loci (the second term in Eq 4), placing mutations on the tree as a Poisson process, invoking Brownian motion, etc. Common practices in both fields—e.g., normalizing genotypes by their heterozygosity or using OU processes—can be motivated by simple models that include selection, but they do not constitute a principled approach to incorporating drift and selection into models of trait covariance. In particular, the considerations that lead to them are not sufficiently general (e.g., normalizing by heterozygosity does not incorporate drift or pleiotropy), and they are sometimes used in combination with maneuvers that arise from incompatible assumptions. Developing more robust evolutionary-genetic models of genetic contributions to trait covariance is a formidable challenge, but it may lead to stronger statistical practices that can be used in both micro- and macroevolutionary studies.

We suspect that there are additional connections between statistical genetics and phylogenetics that we have not mapped out here and that could be profitably explored. For example, in most of the applications in which phylogenomic data are used to inform mapping studies, researchers have large-scale phenotypic and genomic sampling for a focal population or species and then sparser genomic sampling (often a single genome) and an estimate of phenotypic means (if even that) for the others. However, there are emerging data sets from closely related species that have dense phenotypic and genomic samples from multiple lineages [187,188]. We anticipate that our framework could be used to derive more principled and powerful approaches for analyzing these types of data. At the other extreme are methods in which we have sparse sampling of both phenotypes and genomes for a phylogenetically diverse set of species (which generally fall under the PhyloG2P label, mentioned above [60]). In this case, researchers either use phylogenetic data to uncover convergent mutations associated with phenotypic convergence across lineages (e.g., [189]) or more commonly, identify regions with a relatively large number of substitutions—but not necessarily the same ones—in phylogenetically distinct lineages that have convergently evolved the same phenotype [190,191]. For example, Sackton and colleagues [192] used such an approach to identify regulatory regions that had high rates of evolution in lineages of flightless birds; they also demonstrated that some of these regions influence wing development using experimental perturbations. Such rate association tests (see also [193]) seem to be very similar, both conceptually and statistically, to techniques used in rare-variant association studies, which look for local enrichment of rare variants in cases versus controls, rather than associating single variants with phenotype [52,161–163]. We suspect that one could derive a formal equivalence between these sets of methods as we did between GWAS and PGLS above using similar techniques.

There are clear biological rationales explaining why various types of analyses will be more or less informative at different timescales. But this is a difference of degree and not of kind. And the different methodological traditions in statistical genetics and phylogenetics are just that—traditions. There is no reason that researchers should think about the problem of trait mapping in fundamentally distinct ways just because they happened to be trained in a statistical genetics or phylogenetics lab. Ultimately, we should work to take the best ideas from both of these domains and blend them into a more cohesive paradigm that will clarify the molecular bases of phenotypes.

## 4. Materials and methods

### 4.1. Simulation details

To perform phylogenetic simulations, we used the fastBM function from the phytools R package [194]. In all cases, Brownian motions were simulated independently and with rate 1. When performing phylogenetic simulations of Felsenstein’s worst case, we used stree from ape [195] to simulate 2 star trees of 100 tips, where each tip in the star tree had length 0.5. We then connected the 2 star trees using internal branches of varying length. To add a non-Brownian confounder, in each simulation we added an independent normal random variable with varying standard deviations to the *x* and *y* values for individuals from clade 1. (Within a given simulation, all individuals in clade 1 were augmented by the *same* value for each trait, while between simulations, the confounding effect was a random draw.) When performing simulations in a more complicated phylogeny, we used TreeSim [196] to generate pure-birth trees with birth rate = 1 and complete taxon sampling. Each simulation replicate used a different tree. For ordinary least squares on phylogenetic data, we used the R function lm. For PGLS on phylogenetic data, we used the R package phylolm [110] with the Brownian motion model and no environmental noise.

To perform GWAS simulations, we first generated neutral tree sequences and mutations using msprime [115]. To ensure our results were not simply due to genetic linkage, we simulated a high recombination of 10^−5^ per generation with a mutation rate an order of magnitude lower, 10^−6^ per generation. We first simulated causal variants on a sequence of length 100,000 and generated phenotypes by sampling an effect size for each variant from a normal distribution with mean 0 and variance $\frac{0.01}{2p_{l}(1-p_{l})}$, where *p*_*l*_ is the allele frequency of variant *l*. We then created each individual’s phenotype using the additive model, Eq 4. We then added environmental noise so that the trait’s heritability was less than 1. In all simulations, every population had diploid population size 10,000. To simulate the variant being tested for association, we simulated independent tree sequences and mutations and selected a random variant with allele frequency greater than 0.1. When simulating a GWAS analogue of Felsenstein’s worst case, we drew 100 diploid samples from each population and varied the divergence time of the 2 populations. To include an environmental shift in 1 population, we added a normal random variable with varying standard deviation only to individuals in population 1. To simulate under a more complicated population structure, we simulated 20-tip pure birth trees using TreeSim with a birth rate of 5. We then multiplied all branch lengths by 10,000 to convert them into generations and imported them into msprime using the from_species_tree function. We then generated tree sequences and mutations, sampling 10 diploid individuals from each population. Note that each replicate simulation was performed on an independent random population tree. We performed association testing using a custom python implementation of the LMM. We first used restricted maximum likelihood to estimate $\sigma_{G}^{2}$ and $\sigma_{E}^{2}$, followed by using generalized least squares to estimate the regression coefficients and their standard errors.

### 4.2. Phylogenetic analysis of yeast gene expression data

We obtained the species tree, gene expression matrix, and list of physically interacting genes from https://github.com/acope3/GeneExpression_coevolution [121]. We then randomly subsampled 500 genes that had measurements in at least 15 of the 20 species to test for association, resulting in 124,750 pairs. Because of differential missingness among genes, we computed phylogenetic eigenvector loadings only on the subtree for which both genes had data present, meaning that each pair may have had slightly different eigenvector loadings. We then used phylolm [110] with no measurement error to estimate the regression coefficient. For each number of eigenvectors included, we corrected for multiple testing by controlling the FDR at 0.05 using the Benjamini–Hochberg procedure [197].

## Supporting information

S1 Text Complete derivations of results. (PDF)

## Abbreviations

ARG: ancestral recombination graph
eGRM: expected genetic relatedness matrix
GLMM: generalized linear mixed model
GLS: generalized least squares
GRM: genetic relatedness matrix
GWAS: genome-wide association studies
LMM: linear mixed model
MSA: multiple sequence alignment
PCM: phylogenetic comparative method
PGLS: phylogenetic generalized least squares
