# Supplementary material for: Unifying approaches from statistical genetics and phylogenetics for mapping phenotypes in structured populations
Source: PLoS Biol. 2024 Oct 9;22(10):e3002847. doi: 10.1371/journal.pbio.3002847 (PMC11493298; doi:10.1371/journal.pbio.3002847)
Supplement: S1 Text — (PDF) [file pbio.3002847.s001.pdf]

Supporting Information for: Unifying approaches from statistical genetics and phylogenetics for mapping phenotypes in structured populations

Joshua G. Schraiber[1], Michael D. Edge[1,3,*] & Matt Pennell[1,2,3,*]

[1]*Department of Quantitative and Computational Biology, University of Southern California, USA*
[2]*Department of Biological Sciences, University of Southern California, USA*
[3]These authors contributed equally
[*]Corresponding authors: edgem@usc.edu, mpennell@usc.edu

Contents

A The relationship between effect size and allele frequency under strong Gaussian stabilizing selection 2

B Covariance of the genetic component of the phenotype with pedigree-based kinship 3

C Derivation of the eGRM 5

D The eigenvectors of Σ are the projections of the data onto the principal components 7

E The estimated regression coefficient from ordinary least squares with eigenvectors of the covariance matrix as fixed effects 8

F The estimated regression coefficient from generalized least squares 9

Supporting Information

A The relationship between effect size and allele frequency under strong Gaussian stabilizing selection

This derivation follows some of the development leading to equation 14a of Bulmer [1]. The result here is very similar to one first obtained by Latter [2] (see [3, 4] for later discussions) that under assumptions similar to those we make here, the equilibrium genetic variance for a quantitative trait depends on the total mutation rate of alleles that change the phenotype and not on the distribution of effect sizes. That is, the contribution of a mutation to the additive genetic variance does not depend on its effect size.

Under Gaussian stabilizing selection on a single phenotype, the fitness of an individual with phenotype y is given by

$$w(y) \propto e^{-\frac{(y-O)^2}{2V_s}},$$

where O is the optimal phenotype and V_s is the variance of the stabilizing selection kernel (stronger selection is indicated by smaller V_s). Assuming the population mean is near the optimum, an allele with effect size β will evolve according to underdominant dynamics, in which the minor allele is disfavored with with selection coefficient $\frac{\beta^2}{V_s}$, indicating that alleles that cause larger deviations from the optimum are disfavored, and the strength of selection on individual alleles is related to the strength of selection on the phenotype as a whole [5–7]. Mutation between alleles occurs (symmetrically) at rate μ. Thus,

$$\begin{aligned} \frac{dp}{dt} &= -\frac{\beta^2}{V_s}p(1-p)(\frac{1}{2}-p) + \mu(1-p) - \mu p \\ &= -\frac{\beta^2}{V_s}p(1-p)\left(\frac{1}{2}-p\right) + 2\mu\left(\frac{1}{2}-p\right) \\ &= \left(2\mu - \frac{\beta^2}{V_s}p(1-p)\right)\left(\frac{1}{2}-p\right). \end{aligned}$$

To find an equilibrium, set $\frac{dp}{dt} = 0$. Thus, either

$$\frac{1}{2} - p = 0$$

or

$$\left(2\mu - \frac{\beta^2}{V_s}p(1-p)\right) = 0.$$

The first equation corresponds to an unstable equilibrium. Solving the second equation for β^2 yields

$$\beta^2 = \frac{2\mu V_s}{p(1-p)}$$

as desired.

B Covariance of the genetic component of the phenotype with pedigree-based kinship

With a known pedigree but no genotype data, we might derive the covariances among individuals in the genetic component of a trait by fixing the (unobserved) effect sizes and allele frequencies. For simplicity, we will also assume that individuals are not inbred. Given the pedigree, at any given locus, diploid individuals i and j have some probability of inheriting 0, 1, or 2 alleles identical by descent (IBD). Call these probabilities r_0, r_1, and r_2, respectively. For example, for a parent-offspring pair, $r_1 = 1$, and for a pair of full siblings, $r_0 = 1/4$, $r_1 = 1/2$, and $r_2 = 1/4$.

Following equation 4 in the main text, the covariance of the genetic component of the trait for individuals i and j is

$$\mathrm{Cov}(\sum \beta_l G_{il}, \sum \beta_l G_{jl}) = \sum_l \mathrm{Cov}(\beta_l G_{il}, \beta_l G_{jl}) + \sum_{l \neq k} \mathrm{Cov}(\beta_l G_{il}, \beta_k G_{jk})$$

As in the main text, we assume that genotypes at distinct loci are independent, causing the second term to vanish. With the effect sizes treated as fixed constants, we have

$$\mathrm{Cov}\left(\sum \beta_l G_{il}, \sum \beta_l G_{jl}\right) = \sum_l \beta_l^2 \,\mathrm{Cov}(G_{il}, G_{jl}) = \sum_l \beta_l^2 (\mathbb{E}(G_{il} G_{jl}) - \mathbb{E}(G_{il})\mathbb{E}(G_{jl})).$$

The G values are allelic counts of a non-reference allele (we assume the locus is biallelic), and if we fix the non-reference allele frequencies p_l, then $\mathbb{E}(G_{il}) = \mathbb{E}(G_{jl}) = 2p_l$, giving

$$\mathrm{Cov}\left(\sum \beta_l G_{il}, \sum \beta_l G_{jl}\right) = \sum_l \beta_l^2 (\mathbb{E}(G_{il} G_{jl}) - 4p_l^2). \tag{S1}$$

To proceed, we need $\mathbb{E}(G_{il}G_{jl})$ given the IBD probabilities r_0, r_1, and r_2. We thus compute the desired expectation conditional on each IBD state. $G_{il}G_{jl} = 1$ if individuals are heterozygous, $G_{il}G_{jl} = 2$ if one individual is heterozygous and the other is homozygous for the non-reference allele, and $G_{il}Gjl = 4$ if both individuals are homozygous for the non-reference allele. If individuals i and j share no alleles IBD, using Hardy–Weinberg genotype probabilities (per the assumption of no inbreeding), the conditional expectation is therefore

$$\begin{aligned}
\mathbb{E}(G_{il}G_{jl} \mid \mathrm{IBD} = 0) &= 1 \times (2p_l(1-p_l))^2 + 2 \times 4p_l(1-p_l)p_l^2 + 4 \times p^4 \\
&= 4p_l^2(1-p_l)^2 + 8p_l^3(1-p_l) + 4p_l^4 \\
&= 4p_l^2((1-p_l)^2 + 2p_l(1-p_l) + p_l^2) \\
&= 4p_l^2.
\end{aligned}$$

A similar calculation given one allele inherited IBD gives

$$\begin{aligned}\mathbb{E}(G_{il}G_{jl}|\text{IBD}=1) &= 1\times(p_l(1-p_l)^2+p_l^2(1-p_l))+2\times 2p_l^2(1-p_l)+4\times p_l^3\\ &= p_l+3p_l^2.\end{aligned}$$

(To explain the first line: for the first term, the individuals are both heterozygous if the IBD allele is of the non-reference type and the other two are reference alleles, or if the IBD allele is non-reference and both of the non-IBD alleles are reference alleles. For the second term, since one individual is homozygous for the non-reference allele, the IBD allele must be of the non-reference allele, and of the other two alleles, one must be of each type. For the third term, both individuals are homozygous if the IBD allele and both non-IBD alleles are non-reference alleles.)

If the individuals share two alleles IBD, then

$$\begin{aligned}\mathbb{E}(G_{il}G_{jl}|\text{IBD}=2) &= 1\times 2p_l(1-p_l)+4\times p_l^2\\ &= 2p_l+2p_l^2.\end{aligned}$$

Combining these terms weighted by the IBD probabilities gives the desired expectation,

$$\begin{aligned}\mathbb{E}(G_{il}G_{jl}) &= r_0\mathbb{E}(G_{il}G_{jl}|\text{IBD}=0)+r_1\mathbb{E}(G_{il}G_{jl}|\text{IBD}=1)+r_2\mathbb{E}(G_{il}G_{jl}|\text{IBD}=2)\\ &= r_0 4p_l^2+r_1(p_l+3p_l^2)+r_2(2p_l+2p_l^2)\\ &= p_l(r_1+2r_2)+p_l^2(4r_0+3r_1+2r_2)\\ &= 4p_l\theta_{ij}+p_l^2(4-4\theta_{ij}),\end{aligned} \tag{S2}$$

where the last line comes from noticing that $r_0+r_1+r_2=1$ and defining the kinship coefficient $\theta_{ij}=r_1/4+r_2/2$, equal to the probability that a pair of alleles chosen at random, one from individual i and one from individual j, is IBD.

We can now return to the main covariance of interest by plugging the expression for $\mathbb{E}(G_{il}G_{jl})$ from equation S2 into equation S3, giving:

$$\begin{aligned}\text{Cov}\left(\sum\beta_l G_{il},\sum\beta_l G_{jl}\right) &= \sum_l \beta_l^2(\mathbb{E}(G_{il}G_{jl})-4p_l^2)\\ &= \sum_l \beta_l^2(4p_l\theta_{ij}+p_l^2(4-4\theta_{ij})-4p_l^2)\\ &= \sum_l \beta_l^2(4p_l\theta_{ij}-4p_l^2\theta_{ij})\\ &= 2\theta_{ij}\sum_l 2\beta_l^2 p_l(1-p_l)\\ &= 2\theta_{ij}V_A.\end{aligned} \tag{S3}$$

The final line comes from noting that the sum in the previous line is the additive genetic variance (V_A)

under the assumptions here, that is, the variance of the genetic component of the phenotype among outbred individuals. This is the result required in the main text.

C Derivation of the eGRM

If genotypes are independent of effect sizes, we see that

$$\begin{aligned}\operatorname{Cov}(\beta_l G_{il}, \beta_l G_{jl}) &= \mathbb{E}_\beta(\beta_l^2 \mathbb{E}(G_{il}G_{jl} \mid \beta_l)) - \mathbb{E}_\beta(\beta_l \mathbb{E}(G_{il} \mid \beta))\mathbb{E}_\beta(\beta_l \mathbb{E}(G_{jl} \mid \beta)) \\ &= \mathbb{E}(G_{il}G_{jl})\mathbb{E}(\beta_l^2) - \mathbb{E}(G_{il})\mathbb{E}(G_{jl})\mathbb{E}(\beta_l)^2 \\ &= \mathbb{E}(\beta_l^2)\operatorname{Cov}(G_{il}, G_{jl}) + \operatorname{Var}(\beta_l)\mathbb{E}(G_{il})\mathbb{E}(G_{jl}),\end{aligned} \tag{S4}$$

where the second line comes from the assumption that genotypes and effect sizes are independent, and the third line comes from adding and subtracting $\mathbb{E}(G_{il})\mathbb{E}(G_{jl})\mathbb{E}(\beta_l^2)$ to the second line and simplifying. This formula is valid for any distribution of effect sizes. Further making the assumption that $\mathbb{E}(\beta) = 0$,

$$\begin{aligned}\mathbb{E}(\beta_l^2)\operatorname{Cov}(G_{il}, G_{jl}) + \operatorname{Var}(\beta_l)\mathbb{E}(G_{il})\mathbb{E}(G_{jl}) &= \mathbb{E}(\beta_l^2)\operatorname{Cov}(G_{il}, G_{jl}) + \mathbb{E}(\beta_l^2)\mathbb{E}(G_{il})\mathbb{E}(G_{jl}) \\ &= \mathbb{E}(\beta^2)\left(\operatorname{Cov}(G_{il}, G_{jl}) + \mathbb{E}(G_{il})\mathbb{E}(G_{jl})\right) \\ &= \mathbb{E}(\beta^2)\mathbb{E}(G_{il}G_{jl}).\end{aligned}$$

So, if all loci are equivalent

$$\operatorname{Cov}(A_i, A_j) = L\mathbb{E}(\beta^2)\mathbb{E}(G_{il}G_{jl}), \tag{S5}$$

where L is again the number of loci. (Because we have assumed that $\mathbb{E}(\beta) = 0$, the $\mathbb{E}(\beta^2)$ could equivalently be replaced by $\operatorname{Var}(\beta)$.) In theory, the expectation of the product of genotypes can be computed based on coalescent theory [8], focusing on the gene trees that describe the pattern of shared inheritance of alleles rather than the distribution of the alleles themselves. In practice, the gene trees underlying genetic variation in the sample cannot be observed directly. However, developing a coalescent approach provides theoretical understanding in its own right, and it also forms a basis for doing complex trait analyses using estimated genome-wide gene trees [9, 10].

Here we develop the gene-tree-based view using a different derivation from that presented by McVean [8]. For simplicity, we assume haploid genetic data, the extension to diploid data is straight-forward but tedious [8]. This argument depends crucially on the assumption that genetic variation is selectively neutral, as in [8]. Incorporating natural selection into gene-tree based models in full generality requires analysis of the ancestral selection graph [11]. First, let $\mathcal{T}$ be a tree (including branch lengths) and $\mathbb{Q}$ be the measure on tree space induced by the population history. We use measure-theoretic notation here because trees are a combination of a discrete branching structure and continuous branch lengths; the density $d\mathbb{Q}(\mathcal{T})$ can be roughly thought of as the probability of a tree with a given topology and given branch lengths. To model mutation, we assume that, conditional on the genealogy, mutations arise on each branch as a Poisson process with rate μ; thus, given the total branch length in the tree, T, the total number of mutations on the tree is

distributed a Poisson random variable with mean μT. We are interested in modeling unlinked, segregating loci as are commonly assumed to contribute to genetic variation on a trait in statistical genetics. Thus, we condition on trees containing exactly one mutation. (A more general approach would allow for L mutations to occur on the full ancestral recombination graph, regardless of linkage.) To obtain the density of trees in the infinite-sites limit, we send the mutation rate to 0, which can be thought of as sending the length of a genomic region to a single site

$$\begin{aligned} d\mathbb{Q}(\mathcal{T} \mid \text{mutation}) &= \lim_{\mu \downarrow 0} \frac{\mathbb{P}(\text{mutation} \mid \mathcal{T}) d\mathbb{Q}(\mathcal{T})}{\mathbb{P}(\text{mutation})} \\ &= \lim_{\mu \downarrow 0} \frac{\mu T e^{-\mu T} d\mathbb{Q}(\mathcal{T})}{\int_{\mathcal{T}} \mu T e^{-\mu T} d\mathbb{Q}} \\ &= \frac{T d\mathbb{Q}(\mathcal{T})}{\int_{\mathcal{T}} T d\mathbb{Q}(\mathcal{T})} \\ &= \frac{T d\mathbb{Q}(\mathcal{T})}{\mathbb{E}(T)} \end{aligned} \tag{S6}$$

This formula indicates that conditioning on a gene tree having a mutation results in trees with more total branch length than average. Hence, the distribution of gene trees that underlie SNPs is different from the unconditional distribution of gene trees; in particular, gene trees that have a SNP will tend to have more total branch length than those that do not have a SNP.

Next, note that two haploid individuals will have the same genotype at a variable site only if that mutation occurred on a branch that is ancestral to both samples. Letting T_{ij} be the time in branches ancestral to both samples, we have

$$\begin{aligned} \mathbb{E}(G_{il} G_{jl} \mid \mathcal{T}, \text{mutation}) &= \lim_{\mu \downarrow 0} \frac{\mathbb{P}(G_{il} = 1, G_{jl} = 1, \text{mutation} \mid \mathcal{T})}{\mathbb{P}(\text{mutation} \mid \mathcal{T})} \\ &= \lim_{\mu \downarrow 0} \frac{\mu T_{ij} e^{-\mu T_{ij}}}{\mu T e^{-\mu T}} \\ &= \frac{T_{ij}}{T}. \end{aligned} \tag{S7}$$

This fits the intuition that, conditional on a site being variable, individuals will share that mutation only if it

occurs in a common ancestor of those two individuals. Finally, putting these pieces together

$$\begin{aligned}
\mathbb{E}(G_{il}G_{jl} \mid \text{mutation}) &= \mathbb{E}(\mathbb{E}(G_{il}G_{jl} \mid \mathcal{T}, \text{mutation}) \mid \text{mutation}) \\
&= \mathbb{E}\left(\frac{T_{ij}}{T} \mid \text{mutation}\right) \\
&= \int_{\mathcal{T}} \frac{T_{ij}}{T} d\mathbb{Q}(\mathcal{T} \mid \text{mutation}) \\
&= \int_{\mathcal{T}} \frac{T_{ij}}{T} \frac{T d\mathbb{Q}(\mathcal{T})}{\mathbb{E}(T)} \\
&= \int_{\mathcal{T}} \frac{T_{ij}}{\mathbb{E}(T)} d\mathbb{Q}(\mathcal{T}) \\
&= \frac{\mathbb{E}(T_{ij})}{\mathbb{E}(T)},
\end{aligned}$$

so that

$$\text{Cov}(A_i, A_j) = L\mathbb{E}(\beta^2)\frac{\mathbb{E}(T_{ij})}{\mathbb{E}(T)} \tag{S8}$$

We note that this formula explicitly requires conditioning on the site being variable and that we are ignoring the effects of linkage disequilibrium among sites.

D The eigenvectors of Σ are the projections of the data onto the principal components

To see that the eigenvectors of Σ are equivalent to the projections of the data onto the principal components, suppose we have $n \times L$ genotype matrix G whose rows represent individuals and whose columns represent genetic loci. Entry i, j contains the number of copies of a non-reference allele carried by individual i at locus j. Then, the projections of the data onto the kth principal component is given by

$$v_k = Gw_k$$

where w_k is the kth eigenvector of the $L \times L$ covariance matrix of the *genotypes*, i.e. $G^TGw_k = \lambda_k w_k$. Then,

$$\begin{aligned}
G^TGw_k &= \lambda_k w_k \\
\implies \underbrace{GG^T}_{\Sigma}\underbrace{Gw_k}_{v_k} &= \lambda_k \underbrace{Gw_k}_{v_k} \\
\implies \Sigma v_k &= \lambda_k v_k,
\end{aligned}$$

where the second line follows from left multiplying the first line by G. For clarity, we emphasize that this is distinct from computing eigenvectors of the phenotypic matrix (if multiple phenotypes are measured), which is intended to solve a different problem [12, 13]. However, if all traits evolve by identical Brownian motions,

the eigenvectors from the phenotype matrix will coincide with the eigenvectors of Σ.

E The estimated regression coefficient from ordinary least squares with eigenvectors of the covariance matrix as fixed effects

We construct a design matrix $X = \begin{bmatrix} x & v_1 & v_2 & \cdots & v_J \end{bmatrix}$, whose columns are the predictor x, followed by the first J eigenvectors of Σ. (In theory, any set of eigenvectors of Σ might be included in the design matrix, but in statistical-genetic practice, it is typically the leading eigenvectors.) Then, using standard theory, the vector of coefficient estimates is

$$\hat{\beta} = (X^T X)^{-1} X^T y.$$

Then,

$$X^T X = \begin{bmatrix} x^T x & v_1^T x & v_2^T x & \cdots & v_J^T x \\ v_1^T x & 1 & 0 & \cdots & 0 \\ v_2^T x & 0 & 1 & \cdots & 0 \\ \vdots & \vdots & \vdots & \ddots & \vdots \\ v_J^T x & 0 & 0 & \cdots & 1 \end{bmatrix},$$

i.e. the first row and columns are the projection of x along each eigenvector and the rest of the matrix is an identity matrix. The identity matrix arises because the eigenvectors annihilate each other. Then,

$$(X^T X)^{-1} = \frac{1}{x^T x - \sum_{j=1}^{J} (v_j^T x)^2} \begin{bmatrix} 1 & -v_1^T x & -v_2^T x & \cdots & -v_J^T x \\ -v_1^T x & x^T x - \sum_{j \neq 1}^{J} (v_j^T x)^2 & -(v_1^T x)(v_2^T x) & \cdots & -(v_1^T x)(v_J^T x) \\ -v_2^T x & -(v_2^T x)(v_1^T x) & x^T x - \sum_{j \neq 2}^{J} (v_j^T x)^2 & \cdots & -(v_2^T x)(v_J^T x) \\ \vdots & \vdots & \vdots & \ddots & \vdots \\ -v_J^t x & -(v_J^T x)(v_1^T x) & -(v_J^T x)(v_2^T x) & \cdots & x^T x - \sum_{j \neq J}^{J} (v_j^T x)^2 \end{bmatrix}.$$

Ultimately, we only need the first row of $(X^T X)^{-1}$ to estimate the regression coefficient for x. Note that by expanding $x^T x$ in terms of the eigenvectors, we have that

$$\begin{aligned} x^T x - \sum_{j=1}^{J} (v_j^T x)^2 &= \sum_{j=1}^{n} (v_j^T x)(v_j^T x) - \sum_{j=1}^{J} (v_j^T x)^2 \\ &= \sum_{j=J+1}^{n} (v_j^T x)(v_j^T x), \end{aligned}$$

so that the first row of $(X^T X)^{-1}$ is

$$(X^T X)^{-1}_1 = \frac{1}{\sum_{j=J+1}^{n} (v_j^T x)(v_j^T x)} \begin{bmatrix} 1 & -v_1^T x & -v_2^T x & \cdots & -v_J^T x \end{bmatrix}$$

We also compute

$$X^T y = \begin{bmatrix} x^T y \\ v_1^T y \\ v_2^T y \\ \vdots \\ v_J^T y \end{bmatrix}$$

so that

$$\begin{aligned}
(X^T X)_1^{-1} X^T y &= \frac{x^T y - \sum_{j=1}^{J} (v_j^T x)(v_j^T y)}{\sum_{j=J+1}^{n} (v_j^T x)(v_j^T x)} \\
&= \frac{\sum_{j=1}^{n} (v_j^T x)(v_j^T y) - \sum_{j=1}^{J} (v_j^T x)(v_j^T y)}{\sum_{j=J+1}^{n} (v_j^T x)(v_j^T x)} \\
&= \frac{\sum_{j=J+1}^{J} (v_j^T x)(v_j^T y)}{\sum_{j=J+1}^{n} (v_j^T x)(v_j^T x)}
\end{aligned}$$

as desired. Note that when $J = 0$, this recovers the ordinary least squares estimator (equation 15 in main text), as expected.

F The estimated regression coefficient from generalized least squares

The regression coefficient can be estimated via generalized least squares,

$$\hat{\beta}_x^{(GLS)} = (x^T \Sigma^{-1} x)^{-1} x^T \Sigma^{-1} y.$$

Recall that we can diagonalize the covariance matrix Σ as

$$\Sigma = V \Lambda V^T,$$

where $V = \begin{bmatrix} v_1 & v_2 & \cdots & v_n \end{bmatrix}$ is a matrix whose columns are the eigenvectors of Σ and $\Lambda = \text{diag}(\lambda_1, \lambda_2, \ldots, \lambda_n)$ is a diagonal matrix whose entries are the eigenvalues of Σ. If we have design matrix $X = \begin{bmatrix} x & v_1 & v_2 & \cdots & v_J \end{bmatrix}$, we can rewrite the genrealized least square estimator as

$$\begin{aligned}
(X^T \Sigma^{-1} X)^{-1} X^T \Sigma^{-1} y &= (X^T (V \Lambda V^T)^{-1} X)^{-1} X^T (V \Lambda V^T)^{-1} y \\
&= (X^T V \Lambda^{-1} V^T X)^{-1} X^T V \Lambda^{-1} V^T y
\end{aligned}$$

where $\Lambda^{-1} = \text{diag}\left(\frac{1}{\lambda_1}, \frac{1}{\lambda_2}, \ldots, \frac{1}{\lambda_n}\right)$, using the fact that V is an orthonormal basis. Next, compute

$$X^T V = \begin{bmatrix} v_1^T x & v_2^T x & \cdots & v_n^T x \\ 1 & 0 & \cdots & 0 \\ 0 & 1 & \cdots & 0 \\ \vdots & \vdots & \ddots & 0 \end{bmatrix}$$

which is an $(J+1) \times n$ matrix, so the pattern extends $J+1$ rows and the identity elements come from the eigenvectors annihilating each other. Then,

$$X^T V \Lambda^{-1} = \begin{bmatrix} \frac{1}{\lambda_1} v_1^T x & \frac{1}{\lambda_2} v_2^T x & \cdots & \frac{1}{\lambda_n} v_n^T x \\ \frac{1}{\lambda_1} & 0 & \cdots & 0 \\ 0 & \frac{1}{\lambda_2} & \cdots & 0 \\ \vdots & \vdots & \ddots & 0 \end{bmatrix}$$

Note that $V^T X = (X^T V)^T$, so that

$$X^T V \Lambda^{-1} V^T X = \begin{bmatrix} \sum_{j=1}^n \frac{1}{\lambda_i} (v_j^T x)^2 & \frac{1}{\lambda_1} v_1^T x & \frac{1}{\lambda_2} v_2^T x & \cdots & \frac{1}{\lambda_J} v_J^T x \\ \frac{1}{\lambda_1} v_1^T x & \frac{1}{\lambda_1} & 0 & \cdots & 0 \\ \frac{1}{\lambda_2} v_2^T x & 0 & \frac{1}{\lambda_2} & \cdots & 0 \\ \vdots & \vdots & \vdots & \ddots & \vdots \\ \frac{1}{\lambda_J} v_J^T x & 0 & 0 & \cdots & \frac{1}{\lambda_J} \end{bmatrix}.$$

As in the case for ordinary least squares, we will only need the first row of the inverse,

$$(X^T V \Lambda^{-1} V^T X)_1^{-1} = \frac{1}{\sum_{J+1}^n \frac{1}{\lambda_j} (v_j^T x)^2} \begin{bmatrix} 1 & -v_1^T x & -v_2^T x & \cdots & -v_J^T x \end{bmatrix}.$$

Next, compute

$$V^T y = \begin{bmatrix} v_1^T y \\ v_2^T y \\ \vdots \\ v_n^T y \end{bmatrix}$$

so that

$$X^T V \Lambda^{-1} V^T y = \begin{bmatrix} \sum_{j=1}^n \frac{1}{\lambda_i} (v_j^T x)(v_j^T y) \\ \frac{1}{\lambda_1} v_1^T y \\ \frac{1}{\lambda_2} v_2^T y \\ \vdots \\ \frac{1}{\lambda_J} v_J^T y \end{bmatrix}$$

and finally

$$(X^T V\Lambda^{-1}V^T X)_1^{-1} X^T V\Lambda^{-1}V^T y = \frac{\sum_{j=1}^{n}\frac{1}{\lambda_i}(v_j^T x)(v_j^T y) - \sum_{j=1}^{J}\frac{1}{\lambda_j}(v_j^T x)(v_j^T y)}{\sum_{j=J+1}^{n}\frac{1}{\lambda_j}(v_j^T x)(v_J^T x)}$$
$$= \frac{\sum_{j=J+1}^{n}\frac{1}{\lambda_i}(v_j^T x)(v_j^T y)}{\sum_{j=J+1}^{n}\frac{1}{\lambda_j}(v_j^T x)(v_J^T x)}$$

as desired. Setting $J = 0$ recovers the GLS estimator without any eigenvectors included as covariates, as in equation 17 in main text.

References

[1] Bulmer M. Maintenance of genetic variability by mutation–selection balance: a child's guide through the jungle. Genome. 1989;31(2):761–767.

[2] Latter B. Natural selection for an intermediate optimum. Australian Journal of Biological Sciences. 1960;13(1):30–35.

[3] Turelli M. Heritable genetic variation via mutation-selection balance: Lerch's zeta meets the abdominal bristle. Theoretical Population Biology. 1984;25(2):138–193. Available from: https://www.sciencedirect.com/science/article/pii/0040580984900170.

[4] Keightley PD, Hill WG. Quantitative genetic variability maintained by mutation-stabilizing selection balance in finite populations. Genetical Research. 1988;52(1):33–43.

[5] Bulmer M. The effect of selection on genetic variability. The American Naturalist. 1971;105(943):201–211.

[6] Bulmer M. The effect of selection on genetic variability: a simulation study. Genetics Research. 1976;28(2):101–117.

[7] Keightley PD, Hill WG. Quantitative genetic variability maintained by mutation-stabilizing selection balance in finite populations. Genetics Research. 1988;52(1):33–43.

[8] McVean G. A genealogical interpretation of principal components analysis. PLoS genetics. 2009;5(10):e1000686.

[9] Link V, Schraiber JG, Fan C, Dinh B, Mancuso N, Chiang CW, et al. Tree-based QTL mapping with expected local genetic relatedness matrices. bioRxiv. 2023;p. 2023–04.

[10] Zhang BC, Biddanda A, Gunnarsson AF, Cooper F, Palamara PF. Biobank-scale inference of ancestral recombination graphs enables genealogical analysis of complex traits. Nature Genetics. 2023 May;55(5):768–776. Available from: https://doi.org/10.1038/s41588-023-01379-x.

[11] Krone SM, Neuhauser C. Ancestral processes with selection. Theoretical population biology. 1997;51(3):210–237.

[12] Revell LJ. Size-correction and principal components for interspecific comparative studies. Evolution. 2009;63(12):3258–3268.

[13] Uyeda JC, Caetano DS, Pennell MW. Comparative analysis of principal components can be misleading. Systematic Biology. 2015;64(4):677–689.
